# Unraveling the Complexity of Wildland Urban Interface Fires

**DOI:** 10.1038/s41598-018-27215-5

**Published:** 2018-06-18

**Authors:** Hussam Mahmoud, Akshat Chulahwat

**Affiliations:** Department of Civil and Environmental Engineering, Colorado State University, Colorado, CO 80523 USA

## Abstract

Recent wildland urban interface fires have demonstrated the unrelenting destructive nature of these events and have called for an urgent need to address the problem. The Wildfire paradox reinforces the ideology that forest fires are inevitable and are actually beneficial; therefore focus should to be shifted towards minimizing potential losses to communities. This requires the development of vulnerability-based frameworks that can be used to provide holistic understanding of risk. In this study, we devise a probabilistic approach for quantifying community vulnerability to wildfires by applying concepts of graph theory. A directed graph for community in question is developed to model wildfire inside a community by incorporating different fire propagation modes. The model accounts for relevant community-specific characteristics including wind conditions, community layout, individual structural features, and the surrounding wildland vegetation. We calibrate the framework to study the infamous 1991 Oakland fire in an attempt to unravel the complexity of community fires. We use traditional centrality measures to identify critical behavior patterns and to evaluate the effect of fire mitigation strategies. Unlike current practice, the results are shown to be community-specific with substantial dependency of risk on meteorological conditions, environmental factors, and community characteristics and layout.

## Introduction

Wildfire intensity and occurrence rate have risen alarmingly in recent years^1^. The consequences of these wildfires, particularly when interacting with communities, have been dire and have resulted in substantial socio-economic losses all over the world^2,3^. In North America, most notably was the 2016 Mcmurray fire in which the fire burned through 1,500,000 acres, causing destruction of approximately 2,400 homes and forcing an excess of 88,000 people to flee. Classified as the costliest disaster in Canadian history, the corresponding economic losses reached approximately C$9 billion. In the U.S., especially in the west, not only the intensity of wildfires are on the rise^4–6^, but the fire season is elongating as well^7,8^. In 2017, the North Bay fire, which included 14 wildfires across California, burned over 245,000 acres, torched 8,800 homes and claimed the lives of 42 people, making it one of the most deadly wildfires in the history of the state.

As of now, the yearly federal expenditures on managing wildfires easily exceeds US$1 billion per year^1^, and is only expected to rise given the prevalent trend. Current approaches to managing wildfires focus on fire suppression and managing fuel build-up in wildlands. However, reliance on these strategies alone has proven inadequate^9–12^. Currently, wildfire suppression has led to reduction in controlled small-scale fires. This has aided in reducing wildland density and providing an ecological balance. However, in the absence of any large natural reduction mechanism and given the limited fuel management strategies, rapid growth in wildland fuel has resulted in significant increase in high intensity wildfires^10,13^. Wildfires are a part of nature, both inevitable and necessary, pointing to only one foregone conclusion - Wildfire management needs to be driven by regulating vulnerability of communities^10,14^. This includes, but not necessarily limited to, selective wildland fuel treatment at the community interface, reduction of stray vegetation in the house ignition zone, reinforcing households with fire proof methodologies and sustainable urban planning geared towards reduction of fire risk.

There exist several wildfire propagation models that are currently being used by fire management agencies and researchers all over the world. These models encompass different types ranging from empirical to completely analytical. Some prominent examples include - Rothermel’s wildland fuel model^15^, National bushfire model^16^, BehavePlus^17^, FlamMap^18,19^, FARSITE^20^, FSPro^21^, WIFIRE^22^ and others^23,24^. These models are widely accepted and entail several aspects of wildfires; however, the models either entirely focus on wildlands or pertain to a localized aspect of fire propagation in communities. While robust computational fluid dynamic (CFD) models exist for simulating structure-fire interactions on a community scale^25^, their complexity and computational demand prevent their widespread application. The lack of requisite data for wildfires further prevents better understanding and modeling of wildfires let alone their interaction with communities. With advances in computational infrastructure, in the near future, the use of CFD models will become a reality. However, with the risk of WUI fires on an astronomic rise each year, we cannot afford to wait for the technology to match the research requirements. With this in mind, the pressing need lies in exploring alternative directions for quantifying and studying WUI risk of communities. We suggest that a model based on the concept of graph theory would be able to bridge this gap, not only for quantifying the risk but also for unraveling the complexity of these events. Computationally efficient models for fire propagation have been explored in the past using concepts of graph theory, both for wildlands^26–28^ and urban settings^29,30^. These models on urban and wildland fire simulation were based on the concept of minimum travel time. While it is a suitable performance metric, and even necessary for on-site managers, the rate of spread of a wildfire may not necessarily represent its true damage potential. There are other performance metrics, such as community vulnerability, that need to be deliberated especially when considering sustainable urban planning, which have not been previously explored.

For wildfires, researchers have developed detailed frameworks to quantify the potential of fire spread in wildlands; however, there is currently no standardized method of risk assessment that can be applied to WUI communities nationwide^31^. The propagation behavior of WUI fires inside a community can be considered similar to that of systemic transmission of diseases in a social network. Graph theory has been widely utilized to understand disease transmission^32–38^, which has provided unparalleled advances in the field. Similarly, the use of graph theory may be able to provide a better understanding of WUI fire behavior in communities. In this study, a graph model is proposed to evaluate vulnerability of communities to wildfires. The model is first tested on a sample community from California to observe the effect of wind conditions on fire propagation. Followed by tests on Oakland (California), which was ground zero for the infamous 1991 Tunnel Fire. The model is calibrated to conditions similar to the historic Oakland fire and tested to identify the underlying factors affecting community vulnerability to wildfires.

## Graph Theory Model: AGNI-NAR

When a wildfire enters a community it undergoes discontinuous propagation due to discretization of its propagation space. A community comprises of ignitable, as well as, non-ignitable regions, unlike wildlands. Once a wildland fire reaches an urban interface it spreads into the community, propagating from one ignitable source to another (mostly in parallel). The discrete movement of wildfire can be modeled as a flow problem in graph theory. In this study, a quasi-physics-based graph model is presented (AGNI-NAR: ‘*Asynchronous Graph Nexus Infrastructure for Network Assessment of WUI Risk*’), which takes into account the different modes of heat transfer to evaluate propagation probabilities between ignitable components, and subsequently, the vulnerability of a community. Once ignitable areas of the community in question are identified, a suitable directed graph is developed. Each area/structure of a community is defined by multiple nodes that form a boundary, referred to in this study as a‘way’. Even though parts of the area inside a way are ignitable, only the boundary of each way is defined by nodes. Each node does not represent a particular component, but rather a specific area within a way. A node is considered ignitable if even a small part of the area it covers is ignitable. Ideally, a high number of nodes can be used to model different components within a way, however that would increase the computational cost substantially.

**Figure 1:**
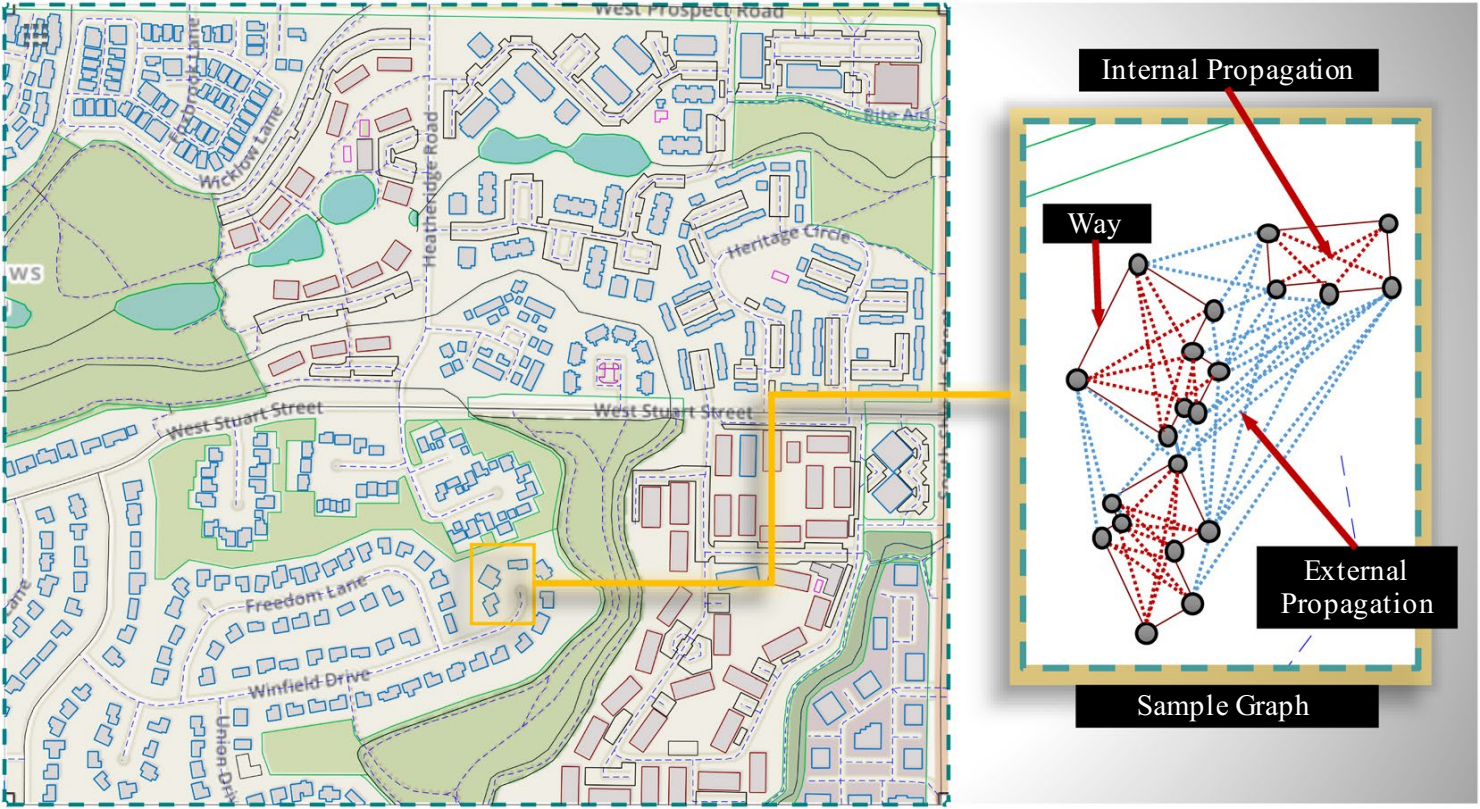
A sample representation of actual community layout (Fort Collins, Colorado, USA) as a graph network. The nodes of each way define its specific boundary and the edges represent the potential fire propagation paths (©OpenStreetMap contributors^55^).

The ignitable ways are utilized to form a directed graph, defined as ${\mathscr{G}}=({\mathscr{V}},{\mathscr{E}})$, where ${\mathscr{V}}=\{{v}_{1},\ldots ,{v}_{n}\}$ defines the node set and ${\mathscr{E}}\subseteq {\mathscr{V}}\times {\mathscr{V}}$ defines the edge set. The adjacency matrix of the respected graph is defined as $A=[{a}_{(i,j)}]\in {{\mathbb{R}}}^{nxn}$ associated with ${\mathscr{G}}$ such that ${a}_{(i,j)}={({P}_{tr}^{(i,j)})}_{\{i\in {\mathbb{Z}},j\in {\mathbb{Z}}\}}$, where ${P}_{tr}^{(i,j)}\in [0,1]$ is the probability of fire transfer from ignitable node *i* to ignitable node *j*. Nodes *i* and *j* are part of ways (same or different), as the boundary of each way is described by node set ${{\mathscr{W}}}_{(m)}$ such that ${\mathscr{V}}={\cup }_{m=1}^{{N}_{w}}{{\mathscr{W}}}_{(m)}$, where ${N}_{{\mathscr{W}}}$ is the total number of ways. A sample graph formulation is shown in Fig. 1 for a segment of the city of Fort Collins (Colorado). GIS data acquisition and classification is discussed in section 1 of the SI text. Fire propagation between nodes is classified into two types, based on the nature of source and target nodes, as - (1) Internal and (2) External propagation (Fig. 1). The former involves propagation within a particular ignitable way and the latter includes propagation from one ignitable way to another. The cumulative fire transfer probability along each edge from node *i* (under ignition) to node *j* is given by Eq. 1, such that internal propagation is governed dominantly by conduction mode^39^, whereas, external propagation is controlled by multiple modes^40^. When nodes *i* and *j* belong to the same way, the ignition transfer probability is given by conduction probability only - ${P}_{cond}^{(i,j)}\in \{0,1\}$. The dependence of conduction propagation on material properties is not considered, hence ${P}_{cond}^{(i,j)}=1$ for all cases. In case of external propagation, 3 modes of heat transfer are considered - (1) Convection - ${P}_{conv}^{(i,j)}\in \{0,1\}$ (2) Thermal radiation - ${P}_{rad}^{(i,j)}\in [0,1]$ and (3) Ember spotting - ${P}_{ember}^{(i,j)}\in [0,1]$, which account for majority of fire propagation in WUI fires^41^. The total probability of external propagation is defined as the union of individual mode probabilities (Eq. 2), which are evaluated based on their respective formulated models (see Material and Methods and sections 2–4 of the SI text).

1 $${P}_{tr}^{(i,j)}=[\begin{array}{cc}min({P}_{total}^{(i,j)},1) & {\rm{i}}{\rm{f}}\,{\{j\notin {{\mathscr{W}}}_{(m)}:i\in {{\mathscr{W}}}_{(m)}\}}_{m\in {\mathbb{Z}}}\\ {P}_{cond}^{(i,j)} & {\rm{i}}{\rm{f}}\,{\{j\in {{\mathscr{W}}}_{(m)}:i\in {{\mathscr{W}}}_{(m)}\}}_{m\in {\mathbb{Z}}}\end{array}$$

2 $${P}_{total}^{(i,j)}=({P}_{conv}^{(i,j)}\cup {P}_{rad}^{(i,j)}\cup {P}_{ember}^{(i,j)})$$

## Vulnerability of Ways

### Framework

The problem of wildfire propagation constitutes a parallel replication flow problem. Depending on the conditions, wildfire propagates both short- and long-range distances at the same time. Given the dependence of both modes on probability of propagation, the vulnerability of any way in the community is defined as the probability of wildfire reaching it. The vulnerability of the destination way is calculated by identifying the Most Probable Paths (MPP) from source to target way (section 5 of the SI text), following the principle of least resistance. Therefore, the total probability of propagation along a MPP is defined as the product of the edge weights (Eq. 3), such that ${ {\mathcal M} }_{(x)}$ is the adjacency list of *x* MPP given by ${ {\mathcal M} }_{(x)}=\{({n}_{(1)}\to {n}_{(2)}),\ldots ,({n}_{({N}_{{ {\mathcal M} }_{(x)}}-1)}\to {n}_{({N}_{{ {\mathcal M} }_{(x)}})})\}$, where ${N}_{{ {\mathcal M} }_{(x)}}$ is the total members in adjacency list ${ {\mathcal M} }_{(x)}$.

3 $${P}_{MPP}^{(x)}=\prod _{(i\to j)\in { {\mathcal M} }_{(x)}}{P}_{tr}^{(i,j)}$$

Following the concept of Flow Centrality, multiple surrogate paths are expected to reach a target way; hence, the total probability is defined as the mean probability of *K* MPPs ${P}_{m}^{(s)}$ (Eq. 4). *K* paths are chosen to account for the closeness amongst the parallel path probabilities. Due to computational limitations, *K* = 10 is used for all analysis in this study. By increasing the number of parallel paths, accuracy of the model can be improved at the cost of increased computation time. Details of the computational cost incurred in this formulation is discussed in section 6 of the SI text. A community layout with high density would require a low *K* value, and vice-versa. It would be the designer’s decision to balance this trade-off.

4 $${P}_{m}^{(s)}=\frac{1}{K}\sum _{x=1}^{K}{P}_{MPP}^{(x)}$$

**Figure 2:**
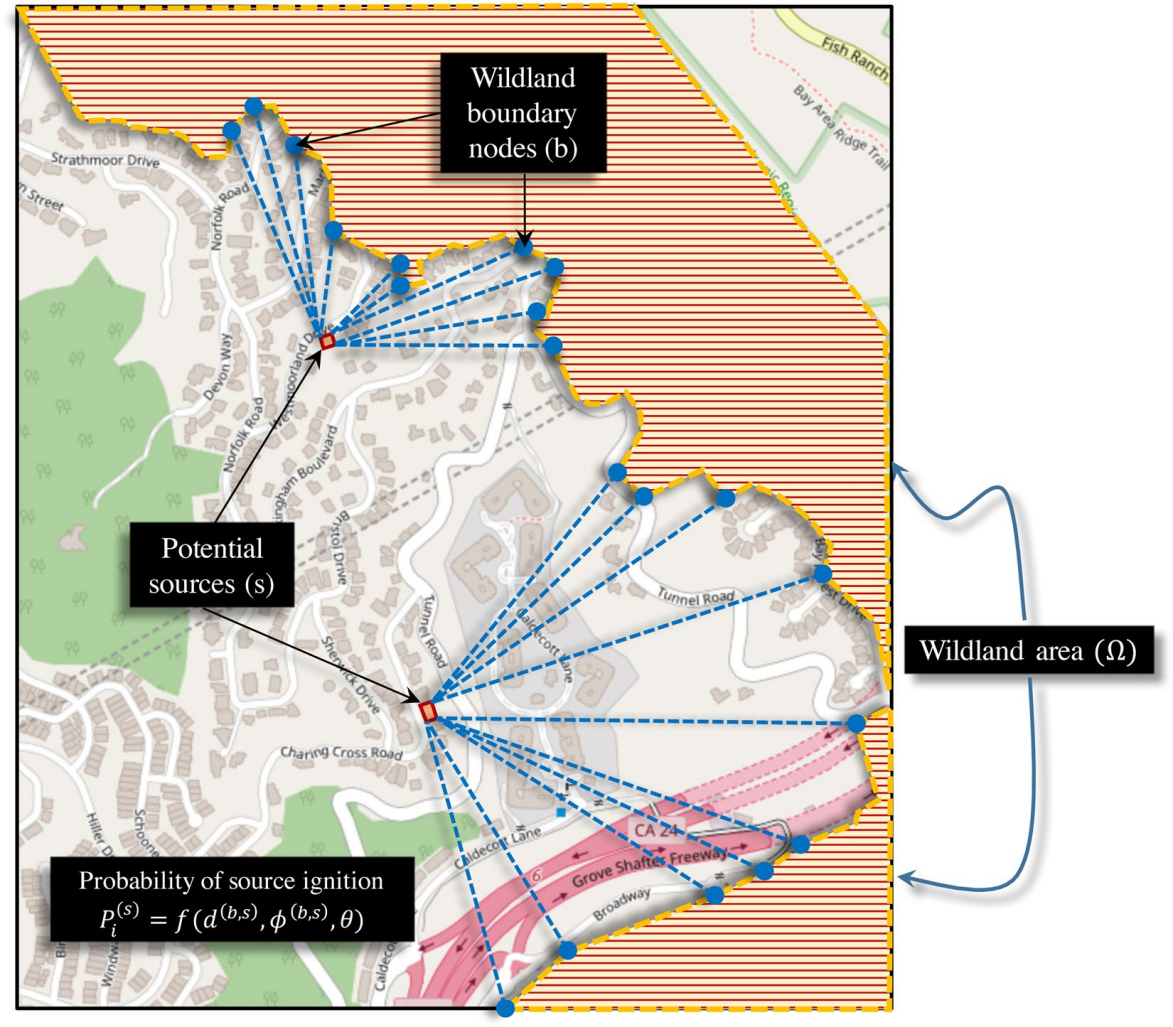
Procedure for evaluating probability of ignition for any source node *s* inside the community due to the wildlands (Ω) (©OpenStreetMap contributors^55^).

A wildfire entering a community has multiple source nodes (initial point of fire origin in the community) at the wildland-urban interface, or some even inside the community, due to ember spotting from wildlands^40,42,43^. To account for this source variability, Eq. 5 is used to calculate the total vulnerability (*V*^(*z*)^) of destination node $z\in {{\mathscr{W}}}_{(m)}$, where ${{\mathscr{W}}}_{m}$ is the node set for way *m*, ${P}_{i}^{(s)}$ is the ignition probability of source node *s* and ${\mathscr{S}}$ is the node set of all sources. The wildfire is required to reach the target from only one of the sources for pilot ignition; hence, the vulnerability is defined by maximum probability from all source nodes. Since probability of ignition for each source is correlated to wind conditions and wildland vegetation in the vicinity of the community (Fig. 2), these conditions need to be considered while evaluating ignition probability for each source.

5 $${V}^{(z)}=\,\mathop{max}\limits_{\{{\rm{s}}\in {\mathscr{S}}\}}\,({P}_{i}^{(s)}.{P}_{m}^{(s)})$$

### Effect of wind direction and speed

**Figure 3:**
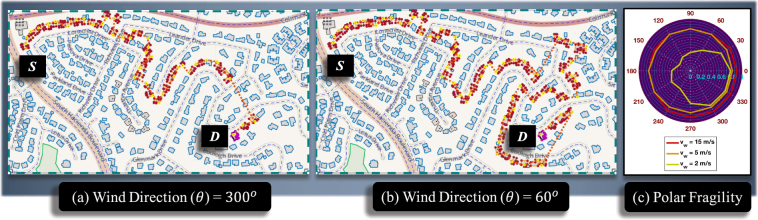
Calculation of vulnerability of destination way *D* due to source way *S* for a part of an actual community Hacienda Heights, Los Angeles, CA. (**a**) Fire pathways shown for wind direction *θ* = 300^*o*^ (**b**) Fire pathway shown for wind direction *θ* = 60^*o*^ (**c**) Fragility curve to represent variation in vulnerability of way *D* for different wind directions (*θ*) and wind speed *v*_*w*_ (©OpenStreetMap contributors^55^).

To assess the effect of wind speed and direction, a part of Hacienda Heights (HH) in Los Angeles (California) is tested. The layout is chosen to represent a typical medium density community. Fire pathways are identified for a target way from a source way for different wind directions (Fig. 3). For wind direction parallel to the line segment joining source and target ways, a high vulnerability is to be expected (*V* = 0.90 for *θ* = 300^*o*^ and *v*_*w*_ = 15 *m*/*s*). However, a relatively high vulnerability is observed for other wind directions as well (Fig. 3(c)). Wind direction is measured anticlockwise from the positive x-axis, such that a N-S wind would be represented by *θ* = 270^*o*^ and S-N wind by *θ* = 90^*o*^. The fire utilized inherent path redundancy present in the community layout to its advantage. The effect of wind direction on vulnerability is seen to be negligible for wind speeds 15 m/s and 5 m/s. In these cases, ember and radiation modes, both, played a dominant role, thereby providing sufficient buffer to each other. A strong dependency of wildfire propagation on community layout is known to exist^42,43^. For low wind speed (*v*_*w*_ = 2 *m*/*s*), wind direction governs vulnerability to a great extent, since the effect of ember and convection modes are reduced. As a result, for wind directions not parallel to line segment connecting the source and target ways, the fire has to rely only on radiation mode. Discontinuities in the community layout inversely affects radiative propagation^43,44^, thereby reducing the vulnerability.

## Community Vulnerability: The Oakland Hills 1991 Wildfire

### Background and modeling details

**Figure 4:**
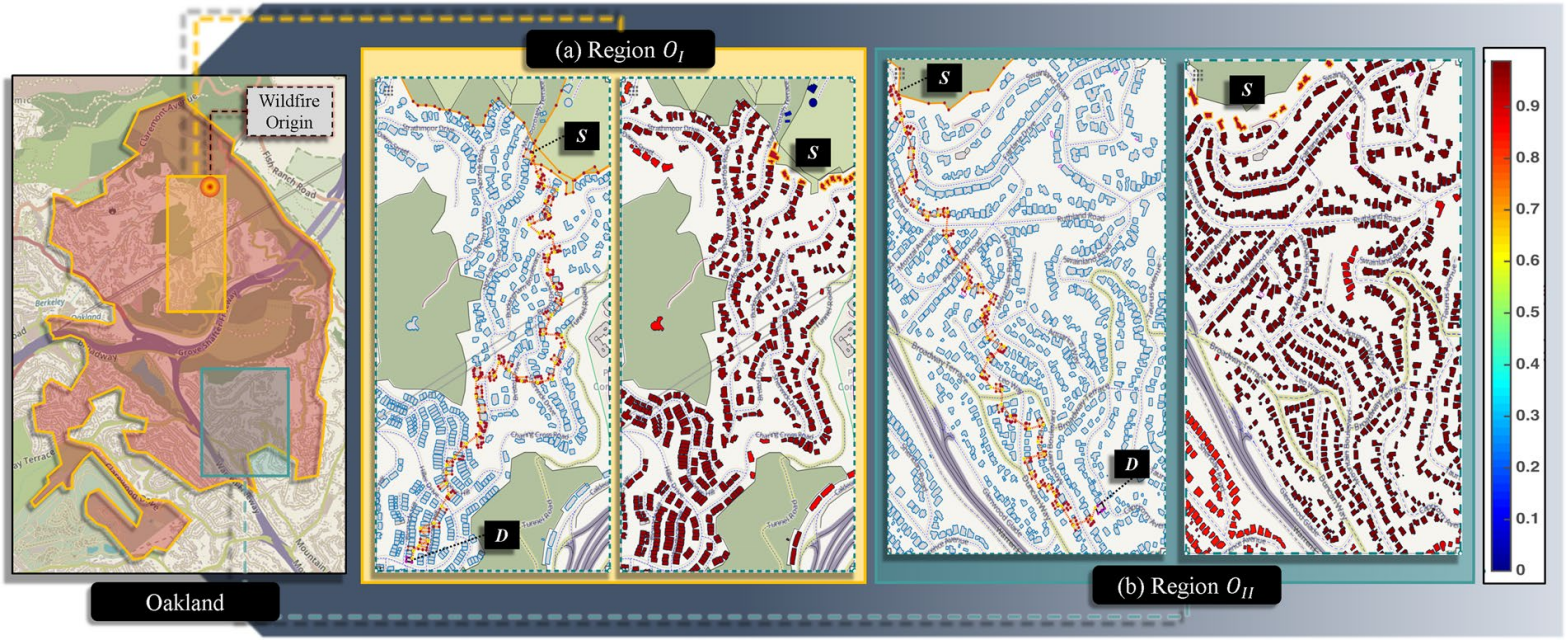
Region of Oakland affected by the 1991 wildfire along with the point of fire origin. Two regions are selected for analysis (**a**) Region *O*_*I*_ with wind direction *θ* = 225^*o*^ and (**b**) Region *O*_*II*_ with wind direction *θ* = 300^*o*^. For both regions, vulnerability for each way is calculated from the source in their respective layouts. Significantly high vulnerability of all ways is observed in both layouts in the absence of any fire mitigation (©OpenStreetMap contributors^55^).

To identify and understand the underlying sources of community vulnerabilities for wildfire, the 1991 Oakland Hills wildfire is considered as a case study. The infamous wildfire, also known as the ‘Tunnel Fire’, resulted in 25 fatalities, 150 casualties and approximately US$ 1.5 billion in economic losses, making it one of the most destructive wildfires in history^45^. Figure 4 shows the origin of the wildfire and the affected region. The fire started as an incompletely extinguished grass fire. However, it quickly escalated to a firestorm when seasonal northerly winds, commonly referred to as ‘Diablo’ winds, entered Oakland hills (at a speed > 100 *km*/*hr*) and reignited the brush fire. The winds propelled the wildfire rapidly in the south-west direction. By noon, the wildfire had crossed two highways and reached Piedmont (South of Oakland hills), after which the winds shifted towards south-east^46,47^. The graph model is tested on Oakland first to understand the level of vulnerability posed to the community in case of a similar event. Second, the model is utilized to identify vulnerability factors of communities to WUI fires. For the tests, conditions similar to that of the 1991 wildfire are simulated. The analysis is performed in two steps by dividing the regions (Fig. 4) into - (a) *O*_*I*_ and (b) *O*_*II*_, for which the wind directions are chosen to be *θ* = 225^*o*^ and *θ* = 300^*o*^, and the wind speed to be *v*_*w*_ = 29.058 *m*/*s*(104.42 *km*/*h*), respectively^46,47^.

### Community inherent vulnerability

The respective vulnerability maps obtained for both regions of Oakland are shown in Fig. 4. The total vulnerability, which is calculated as the mean vulnerability of all ignitable ways in the community, for region *O*_*I*_ is observed to be *V* = 0.9772 and for region *O*_*II*_ to be *V* = 0.9815. Without any form of fire mitigation, internal or external, the vulnerability of wildfire propagation is found to be sufficiently high for both regions of Oakland. This can be attributed to high path redundancy present in the communities due to significant clustering of ways and absence of discontinuities. The vulnerability of HH for *θ* = 300^*o*^ (maximum effect seen for this direction) and *v*_*w*_ = 29.058 *m*/*s* is calculated to be *V* = 0.9326. Even though the fuel density *I* (section 1 of the SI text) of region *O*_*I*_ is lower than HH (Table S2), the vulnerability for *O*_*I*_ is relatively the highest. The layout structure of communities, indeed, plays a role in determining the level of vulnerability of communities^48^.

### Influence of individual ways

To better understand the effect of layout characteristics of a community on fire propagation, the importance of individual ways is classified. Primarily, ways which assert global influence on the wildfire network are identified using Eigenvector centrality (section 7 of the SI text). The most influential ways are observed at the wildland-urban interface (Fig. S2(a)) and the position of these ways changed in correlation to the wind direction (Fig. S2(b) and (c)). This can be explained by high wind speed conditions that allow the ways at the interface to connect to maximum ways by dispersing embers in the direction of wind. Due to high directionality in the graph, the edge nodes have the highest contribution to the network. For the 1991 Oakland wildfire, the location of origin of fire (Fig. 4) coincided with the highest centrality ways (Fig. S2(a)) that probably maximized the spread capacity of the wildfire. The global ways ignite other ways, which further distribute the fire throughout the community. This transition is observed by evaluating Bonacich centrality^49^ (Fig. S3) for different values of attenuation factor *β* ∈ [0, 1], such that *β* = 1 corresponds to eigenvector centrality and *β* = 0 corresponds to degree centrality. A uniform spread pattern is seen for *O*_*II*_, which is to be expected due to high ember dispersal capacity of all ways under high speed winds and homogeneous density of the community.

To better realize the participation role of each way, total degree centrality^50^ is calculated (section 8 of the SI text). The effect of ember mode dominates over the short range modes (convection and radiation) due to high wind speed and a large number of ways contributes equally to ember dispersion (Figs S4(a) and S5(a)). Most houses in Oakland during the 1991 fire comprised of wooden shingle roof, which were identified as the main factor that led to significant increase in ember generation^51^. As a result, the embers completely overwhelmed all suppressive actions and spread rapidly. Therefore, degree centrality is clearly related to wind speed. The total degree centrality is observed to decline with decrease in wind speed and the centrality of ways also shifts (Figs S4 and S5). The correlation (Kendall rank) of degree centrality is observed to reduce for region *O*_*I*_ (Table S8) and *O*_*II*_ (Table S9) as the difference in wind speed increases. This highlights the fact that ways which contribute more for a particular mode might not be significant contributors with respect to other modes. To develop effective fire mitigation strategies, the ways would need to be classified for each mode separately.

The transitivity (section 9 of the SI text), also commonly know as clustering coefficient, is a measure of the ability of the nodes to form triplets. When transitivity is applied to both regions of Oakland no clear pattern is observed (Figs S6(a) and S7(a)). At high wind speeds, since the ember mode is dominant, each way is able to form triplets easily. As a result, a uniform distribution of centrality is observed. When transitivity is applied to both regions without the effect of wind (Figs S6(b) and S7(b)), an interesting pattern is observed. That is, ways with high volume and situated in high density regions exhibited high transitivity, which is to be expected as they have higher capacity to spread fire. However, in region *O*_*II*_, ways with significantly low area situated close to other larger ways also showed high transitivity (Fig. S7(b)). It has already been well established that low intensity exposure from easily ignitable objects, such as garbage, mulch and vegetation, in the vicinity of households tend to maximize the probability of ignition^10,14,42,52^. The ordered set of ways with area smaller than 10th percentile yields mean transitivity of 0.3620 for region *O*_*I*_ and 0.3928 for region *O*_*II*_, which corresponds to 86th and 90th percentile of the transitivity distributions observed, indicating high transmission capacity of low area ways.

### Effect of fire intervention

Fire intervention (mitigation) is characterized by two components - (1) Intervention strength (*μ*), which is related to number of ways under the effect of some form of fire mitigation, and (2) Strategy efficiency (*η*), which is related to the change in vulnerability as the location of ways under fire mitigation are altered. To observe the effect of these factors, the graph model is extended to include a static fire intervention framework (section 10 of the SI text). The inflow (indegree) and outflow (outdegree) of randomly selected ways are altered to induce the effect of fire intervention factors, including but not limited to, action of firefighters, individual structural properties and passive fire mitigation approaches. The framework is implemented on both regions of Oakland *O*_*I*_ and *O*_*II*_ for *N* = 100 iterations and *μ* ∈ [0, 100%] under original wind conditions (*v*_*w*_ = 29.058 *m*/*s*), to obtain respective vulnerability distributions (Fig. S8(a) and S8(b)). Intervention strength has a direct impact on vulnerability, which reaffirms the theory that management of WUI fires is only possible by regulating fire at the individual structural level^5,10,43,48,53^. The mean vulnerability for different *μ* values is observed to be in range [0.089, 0.977] for region *O*_*I*_ and [0.049, 0982] for region *O*_*II*_. Fire intervention is observed to be more effective for region *O*_*I*_ than region *O*_*II*_, which can be attributed to the difference in topographic features of the region layouts. Region *O*_*I*_ has a relatively constricted layout (less redundant paths), whereas, the layout of region *O*_*II*_ is more spread out (more redundant paths). For each intervention strength *μ*, some strategies (iterations) provide better resistance from wildfire than others, suggesting the importance of strategy efficiency. Depending on the location of ways chosen for intervention, a spread in vulnerability (*η*) is observed. Therefore, given a particular community layout, optimal fire intervention configurations can be evaluated to minimize the effect of wildfires on communities.

**Figure 5:**
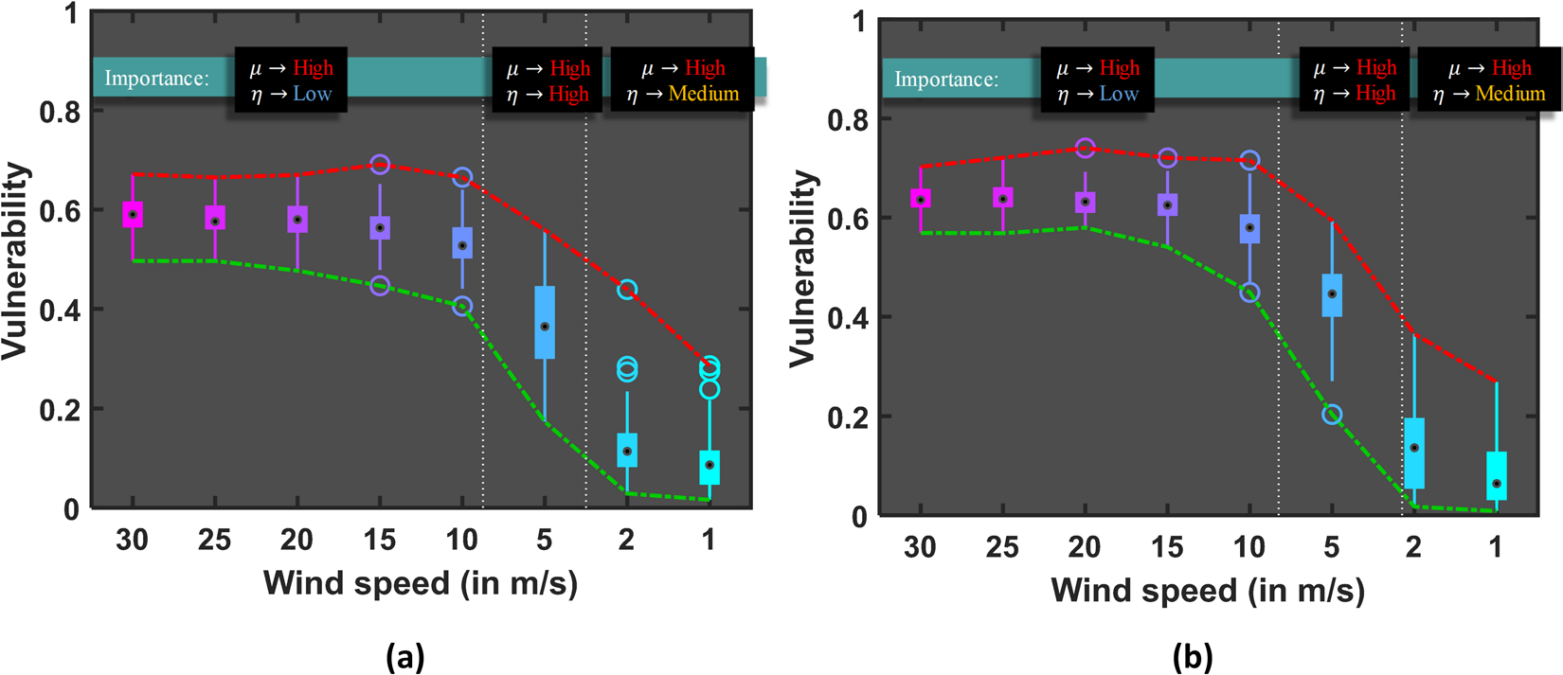
Effect of fire intervention (mitigation) showed for (**a**) region *O*_*I*_ and (**b**) region *O*_*II*_ of Oakland at intervention strength *μ* = 50% for different wind speeds to highlight significance of intervention strategy. Vulnerability distributions are calculated for *N* = 100 iterations at each wind speed and the efficiency of strategy *η* is measured as the standard deviation for each distribution.

During the Oakland fire, all suppressive actions taken by firefighters proved ineffective throughout the day. It was not until the evening, where wind speed gradually reduced and at some point stopped completely, when firefighters were able to stop the fire^46,47^. The intervention framework is applied to both regions at different wind speeds for *μ* = 50% (Fig. 5). At high wind speeds (*v*_*w*_ = [15, 30] m/s), the effect of intervention is nearly constant for both regions, followed by improvement for medium wind speeds. For low wind speeds, the effect is quite significant to the point that some of the configurations result in near zero vulnerabilities. This explains how firefighters were ultimately able to control the 1991 Oakland fire. Strong correlation between wind speed and intervention strength (Fig. S8(a) and (b)) is to be expected. Interestingly, the effect of strategy, which is measured as the standard deviation of vulnerability distribution (*η*), is observed to be maximum for specific range of wind speeds (Fig. 5).

### Effect of source location

All analysis conducted to this point only considered sources at the wildland urban interface (Fig. 4). During the 1991 fire, high speed winds resulted in ignition of several structures inside the community by embers generated from the wildlands, which is a familiar observation in high intensity WUI fires^42,51^. To observe the effects of these internal sources, a framework is developed and implemented (Section 11 of the SI text). For both regions of Oakland, the framework is applied for certain iterations, such that the increase in vulnerability is reduced to less than 1% for further iterations. Specific configurations for fire intervention strength *μ* = 50% are selected for both regions, such that the initial corresponding vulnerabilities (before applying the framework) are calculated to be *V* = 0.541 for region *O*_*I*_ and *V* = 0.610 for region *O*_*II*_. The maximum vulnerability (after 5 iterations) is evaluated to be *V* = 0.774 for region *O*_*I*_ and *V* = 0.733 for region *O*_*II*_. Even though the effect of fire intervention is evaluated to be higher for region *O*_*I*_ than region *O*_*II*_, the effect of additional sources result in higher vulnerability for the former region. Region *O*_*I*_ has a higher percentage of wildland vegetation surrounding it than region *O*_*II*_, as a result, the wildlands are able to generate internal sources further deep into the community. During the first hour of the Oakland fire, a similar pattern of internal sources was observed (Fig. S9) and the vulnerability for this configuration is calculated to be *V* = 0.7933. Two of the actual observed internal source locations coincide with the optimal internal source locations calculated from the framework (Fig. S10(e)). The internal source framework shows that just by igniting five critical ways in the community, the vulnerability can be increased significantly even after considering 50% intervention strength. Wildlands surrounding communities exasperate the problem by creating additional internal sources and these need to be accounted for proper community risk assessment^48^.

### Identifying flow paths

The flow paths of radiation and convection modes are strictly dependent on community layout, unlike ember paths, which are more or less independent of community layout at high wind speeds. Instead, they are function of individual way properties. Betweenness centrality is used to identify most probable paths (MPP) within the communities for short range propagation modes (radiation and convection) (section 12 of the SI text). The identified paths for both regions of Oakland and region of Hacienda Heights (for *θ* = 300^*o*^) are shown in Fig. S12. These are identified by selecting ways with centrality above a certain threshold (${C}_{b\ast }^{w} > 0.075$). The betweenness values are highest for HH, followed by Oakland region *O*_*I*_, and finally region *O*_*II*_. High centrality values for HH (${C}_{b\ast }^{w}\in [0,0.84]$) suggest higher use of flow paths, which coincide with the fact that the intervention framework has the most impact on HH (*V*(*μ* = 50%) = 0.403 for *θ* = 300^*o*^) among the 3 test regions. On the other hand, region *O*_*II*_ shows lowest centrality values (${C}_{b\ast }^{w}\in [0,0.73]$), which implies that the wildfire spread throughout the community via multiple paths in the region. This indicates a closeness among path probabilities; hence, intervention had the least effect, as the fire is able to find surrogate paths much easily. Identification of high probability paths can aid in improving the efficiency of fire mitigation strategies.

## Discussion

In this study, we presented a quasi-physics based graph theory model to assess vulnerability of communities to wildfire, using four key modes of heat transfer. Sub-models for each mode were established based on previous literature on wildfire models. Each mode has certain complexity associated with their mechanism. As a result, certain assumptions had to be adopted for each sub-model, which have been discussed in their subsequent sections in the SI text. Given the preliminary nature of the current study, the sub-models proved sufficient. However, each of them could be either improved or suitably replaced as desired. Lack of data on certain input parameters could further hinder wide spread application of the proposed model; however the model can still be utilized for general theoretical investigations of WUI fires. For instance, sensitivity of parameters to global community behavior varies significantly, thus, some are more dominant than others. An extensive sensitivity analysis can provide an idea of the prominent variables, which would aid in effective dimensionality reduction of the model.

The graph model was tested on two communities - Hacienda Heights and Oakland (California, USA). One of the primary reasons for choosing communities in California was the availability of data (albeit limited) for these regions. The first test was conducted to investigate the correlation between vulnerability of ways, community layout and wind conditions. The second test was conducted to simulate the infamous 1991 Oakland wildfire to identify underlying factors governing fire propagation within a community. Vulnerability of the community was first calculated without the effect of any form of fire intervention to observe the effect of community layout. The next test focused on the effect of individual ways on community vulnerability, which involved the use of centrality measures from graph theory. To simulate the effect of fire intervention strategies a framework was implemented. The analysis conducted was static in nature. As a result, time-dependent behavior of firefighters was not included. While the static model provides a good estimation of effect of passive fire intervention strategies, a dynamic model would be required to accurately capture the behavior of firefighters (active fire intervention). The implemented intervention framework assumed an unreasonably high efficiency of firefighters. The intervention analysis was conducted under varying intervention strength and wind speed to observe their respective correlation with vulnerability. The next analysis focused on the effect of wildlands on community vulnerability. Critical locations in the community were identified, such that when ignited, the vulnerability of a community would be maximized.

There are both, epistemic and aleatoric uncertainties, associated with the current model. The former relates to the simplifications considered in the model and the latter to the absence of relevant data required to calibrate the necessary variables. Both aspects require sufficient work before widespread application of the proposed model can be realized without prejudice. However, the model provides a direction in fire research that might open opportunities to better understand the mechanics of wildland urban interface fires.

## Conclusions

The observations from the analysis conducted in this study reaffirmed some of the long stated findings from previous studies. We were able to quantify and understand the effects of intrinsic factors, which relate to features that are naturally present in a community, and mitigation factors, which can be altered to regulate vulnerability of the community. The intrinsic factors were primarily identified as - (1) Wind speed (2) Wind direction (3) Community layout and (4) Wildland vegetation in the vicinity of the communities. High speed winds were found to strongly affect the generation of embers, which allowed the fire to spread easily. The discontinuities in the community layouts adversely affected fire propagation; however, the effect was minimal for high wind speeds. The wildlands surrounding the communities led to the creation of additional sources in the interior of communities, which caused significant increase in vulnerability. This increase was, however, a function of which structures the embers ignited. By igniting certain structures in the community, the probability of wildfire propagation can be substantially increased. Certain structures have higher geographical contribution to fire spread than others. Thus, by reverse engineering we can determine the boundary limits of wildland vegetation that would minimize this effect. Given the limited resources, a selective fuel treatment approach would both be practical and economical.

To further manage community vulnerability, mitigation factors such as presence of stray vegetation inside communities, layout and material properties of individual structures, and resources available for fire suppression, are critical. The effect of these factors was quantified in terms of mitigation strength, which was measured as the number of houses under some form of fire mitigation, and strategy efficiency, which was related to the selection of such houses. Both components were found to be strongly correlated to vulnerability; however, the effect of strategy was restricted to low to mid-range wind speeds only. Furthermore, depending on the wind conditions during a WUI fire, the community needs to have a certain level of resilience at the individual structural level, in order to have a fighting chance. These measures could include integration of fire-proof materials in household design, adoption of automatic sprinkler systems and/or management of stray vegetation in the household ignition zone. Essentially, a major responsibility of fire management lies within the hands of home owners; therefore, programs such as Fire Adapted Communities, Fire Adapted Communities Learning Network, Firewise Communities USA, and FireSmart Canada need to be implemented rigorously, which is going to require a paradigm shift in current fire management policies.

This study was an attempt to start a dialogue in a new direction of quantifying wildfire risk to communities. Even though the presented graph model is in its stage of infancy, it is our expectation that application of graph theory can be further extended to study other aspects of fire management, including but not limited to, sustainable urban planning, optimal firefighter mitigation strategies and optimal reinforcement of individual structures against fires. By quantifying these aspects, changes in fire policies can be approached in a systematic fashion as it will allow policy makers, planners, and resource managers to develop long-term solutions to make communities more adaptable to wildfires. Specific design guidelines can be established, similar to design philosophies for other hazards, that are exclusive to each community and account for the mentioned critical variables.

## Material and Methods

There are four primary modes of heat transfer from one ignitable way to another that are observed during WUI fires. The four modes are incorporated using repertoire of information from previous research studies on wildfire. The scope of this study is to understand wildfire propagation on a community scale. It is observed from preliminary tests of the case study considered that the effect of internal propagation of ways on the global vulnerability of the community is minimal; hence, conduction mode is modeled in a simplified manner (Eq. 1). The details for the other three models are discussed below.

### Convection

Convection is the transfer of heat from one location to another by movement of fluids. In this study, heat transfer by convection corresponds to ignition of an object due to direct influence of flames. The probability of convection is defined by Eq. 6, which is unity if the distance between nodes *i* and *j* (d^(i,j)^) is within the convection threshold distance (${d}_{conv}^{(i,j)}$), and zero otherwise. It is reasonable to assume that if the flames touch an ignitable object the probability of ignition would be unity.

6 $${P}_{conv}^{(i,j)}=[\begin{array}{cc}1 & {\rm{i}}{\rm{f}}\,\,{d}^{(i,j)}\le {d}_{conv}^{(i,j)}\\ 0 & {\rm{i}}{\rm{f}}\,\,{d}^{(i,j)} > {d}_{conv}^{(i,j)}\end{array}$$

The threshold convection distance is defined as function of flame height, ${h}_{f}^{(i)}$, flame angle, *θ*_*f*_ ∈ [0, 90^*o*^], and wind direction *θ* ∈ [0, 360^*o*^]. Equation 7 defines the convection distance model, which includes the effect of uncertainty in wind direction in the form of a wind correlation coefficient ${F}_{cc}^{(i,j)}\in \mathrm{[0},\,\mathrm{1]}$, given by Eq. 8. The coefficient attains a maximum value of unity when there is perfect correlation between the wind direction and the direction of edge from node *i* to *j* (*ϕ*^(*i*, *j*)^). The wind correlation coefficient is a measure of the uncertainty associated with local changes in wind direction. Details on flame length and the parameters for the model are described in Section 2 of SI text.

7 $${d}_{conv}^{(i,j)}={F}_{cc}^{(i,j)}.{h}_{f}^{(i)}.tan({\theta }_{f})$$

8 $${F}_{cc}^{(i,j)}=[\begin{array}{cc}cos(|{\varphi }^{(i,j)}-\theta |) & {\rm{i}}{\rm{f}}\,\,|{\varphi }^{(i,j)}-\theta | < {90}^{o}\\ 0 & {\rm{i}}{\rm{f}}\,\,|{\varphi }^{(i,j)}-\theta |\ge {90}^{o}\end{array}$$

### Radiation

In this study, the effect of shape of each way on thermal radiation transmitted is considered and each source-target way pair interactions are evaluated individually. Thermal radiation incident flux on a surface $l\in { {\mathcal F} }_{(n)}=\mathrm{\{1,}\ldots ,l,\ldots ,{N}_{l}\}$ of a way due to a burning surface $k\in { {\mathcal F} }_{(m)}=\{\mathrm{1,}\ldots ,k,\ldots ,{N}_{k}\}$ is calculated by the Stefan-Boltzmann law, where the sets ${{\mathscr{F}}}_{(m)}$ and ${ {\mathcal F} }_{(n)}$ represent the surfaces of ways *m* and *n*, *N*_*k*_ and *N*_*l*_ are the total number of surfaces of respective ways. The incident thermal radiation flux (${q}_{(k,l)}^{(m,n)}$) is defined by Eq. 9, where ${A}_{(k)}^{(m)}$ is the radiative area of a surface, $v{f}_{(k,l)}^{(m,n)}$ is the view factor from the source to target surface, *σ* is the Boltzmann constant, ${\varepsilon }_{(k)}^{(m)}$ is the emissivity of source surface, *T*_*f*_ is the flame temperature and *T*_*a*_ is the temperature of the surroundings. For all analysis in the study, all households are assumed to be made entirely of wood, and thus, completely ignitable. ${A}_{(k)}^{(m)}$ is assumed as the total area of surface *k*. In the presence of windows, this might not be the case since once the interior of a structure starts burning the only way for the heat flux to escape would be the window openings on the surfaces of structure. Specific radiative area of each surface would have to be calculated separately, depending on the nature of the way. In real life scenarios, each structure cannot be assumed a single homogeneous source of thermal radiation. Each surface of an ignited structure is a potential source of radiation with different properties, such as surface area and inclination. To better understand the radiation heat transfer between different ways, the boundary of each way is discretized into multiple surfaces, which generate independent heat flux on individual surfaces of the target way. Thus, the cumulative incident heat radiation for each target surface is function of the inclination of the source surface (${{\rm{\Theta }}}_{(k)}^{(m)}$), target surface (${{\rm{\Theta }}}_{(l)}^{(n)}$) and the distance between the surfaces (${d}_{(k,l)}^{(m,n)}$). This is reflected by the view factors ($v{f}_{(k,l)}^{(m,n)}$) calculated using Eq. 10. The view factors are calculated for each source-target surface pairs exclusively using the procedure discussed in Section 3.1 of SI text.

9 $${q}_{(k,l)}^{(m,n)}={({A}_{(k)}^{(m)}.v{f}_{(k,l)}^{(m,n)}.\sigma .{\varepsilon }_{(k)}^{(m)}\mathrm{.((}{T}_{f}{)}^{4}-{({T}_{a})}^{4}))}_{\{k\in { {\mathcal F} }_{(m)},l\in { {\mathcal F} }_{(n)}\}}$$

10 $$v{f}_{(k,l)}^{(m,n)}=\frac{1}{{A}_{(k)}^{(m)}}{\int }_{{A}_{(l)}^{(n)}}{\int }_{{A}_{(k)}^{(m)}}\frac{cos{{\rm{\Theta }}}_{(k)}^{(m)}.cos{{\rm{\Theta }}}_{(l)}^{(n)}}{\pi {({d}_{(k,l)}^{(m,n)})}^{2}}d{A}_{(k)}^{(m)}.d{A}_{(l)}^{(n)}$$

**Figure 6:**
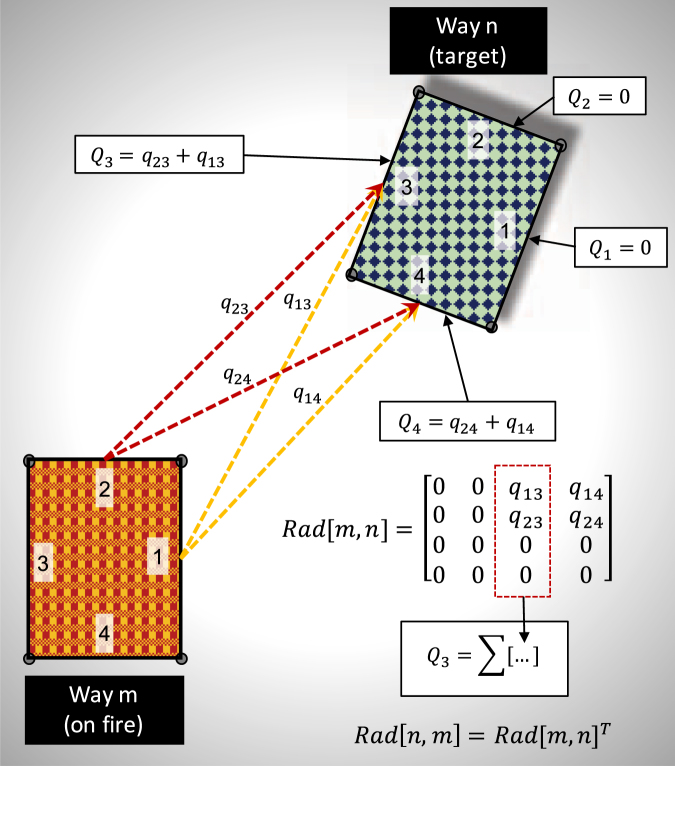
Procedure shown for calculating net radiative heat flux exchange between two ways such that way *m* is ignited. Heat flux exchange between all possible surface pair interactions of ways *m* and *n* are calculated to formulate a radiation matrix that is further used to calculate the net flux received by each surface of way *n*.

Figure 6 shows the thermal radiation interaction between two ways, where way 1 is considered as the ignited source and way 2 as the target. The difference in view angles between the ways generate varied cumulative heat flux on each target way surface. To quantify the effects of radiation between two ways *m* and *n*, a local radiation matrix is defined (*Rad*[(*m*, *n*)]) of size [*k* × *l*] such that each entry represents the net thermal flux exchange between each possible source-target surface combination. The total radiation on the *l*^*th*^ surface of the target way is obtained as the summation along the rows of the *l*^*th*^ column of the radiation matrix. Eq. 11 describes the total incident thermal radiation on surface *l* of way *n* due to way *m* for all *k* surfaces. In case, the roles of ways 1 and 2 are reversed, the modified radiation matrix is directly calculated as *Rad*[1, 2] = *Rad*[1, 2]^*T*^. This result is derived based on the reciprocity rule of heat radiation between 2 surfaces, given as ${A}_{(k)}.v{f}_{(k,l)}^{(m,n)}={A}_{(l)}.v{f}_{(l,k)}^{(n,m)}$. The detailed procedure for formulating the radiation matrix is discussed in Section 3.2 of SI text.

11 $${Q}_{(l)}^{(m,n)}={(\sum ^{k\in {{\mathscr{F}}}_{(m)}}{q}_{(k,l)}^{(m,n)})}_{\{l\in {{\mathscr{F}}}_{(n)}\}}$$

The total incident radiation for surface *l* is utilized to obtain the minimum residence time for flames (${t}_{(l)}^{(m,n)}$) required for ignition. The residence time is defined as the time for which the flames continue to emit heat flux. Depending on the incident radiation heat flux, the flame is required to burn for a minimum residence time before it can cause ignition, and this time is given by Eq. 12 ^39^, where *FTP*^(*n*)^ is the flux time product of the material of target way *n*, ${Q}_{(l)}^{(m,n)}$ is the total incident radiation on surface *l* (as calculated above), ${Q}_{cr}^{(n)}$ is the critical flux required for ignition as a function of target way and *c*^(*n*)^ is a constant derived based on material properties of the target way. The total incident flux is required to be higher than the critical flux for pilot ignition to occur. The parameter values for the model are discussed in Section 3.3 of SI text.

12 $${t}_{(l)}^{(m,n)}=[\begin{array}{cc}FT{P}^{(n)}.({Q}_{(l)}^{(m,n)}-{Q}_{cr}^{(n)}{)}^{c} & {\rm{i}}{\rm{f}}\,{Q}_{(l)}^{(m,n)} > {Q}_{cr}^{(n)}\\ {\rm{\infty }} & {\rm{o}}{\rm{t}}{\rm{h}}{\rm{e}}{\rm{r}}{\rm{w}}{\rm{i}}{\rm{s}}{\rm{e}}\end{array}$$

Based on the minimum ignition time, probability of ignition due to radiation for surface *l* (${p}_{(l)}^{(m,n)}$) is calculated using Eq. 13, where ${t}_{r}^{(m)}$ is the residence time of each surface of source way *m* and $F\mathrm{(.)}:\,{\mathbb{R}}\mapsto \{0,\,1\}$ represents a cumulative density function (CDF) of a normal distribution ${\mathscr{N}}(\mu ,{\sigma }^{2})$. The CDF is used to account for uncertainties associated with residence time of the source way. Eq. 14 gives the radiation ignition probability (${P}_{rad}^{(m,n)}$) between ways *m* and *n* as the maximum of all surface probabilities of target way. The effects of radiation within a community are restricted to a specific distance, as seen from wildfire studies^39,40,43^. A threshold radiation distance (*d*_*th*_) is introduced to ensure ways at a greater distance than the threshold distance are not affected by radiation. The distance ${d}_{min}^{(m,n)}$ is the minimum distance between all possible node combinations of ways *m* and *n*, as given by Eq. 15. The functions max(.) and min(.) correspond to the highest and lowest values in a set/matrix. The mean residence time is calculated as *μ* = (*t*_*r*,*min*_ + *t*_*r*,*max*_)/2.

13 $${p}_{(l)}^{(m,n)}=[\begin{array}{cc}1-F({t}_{r}^{(m)}-{t}_{(l)}^{(n)}) & {\rm{i}}{\rm{f}}\,{t}_{{\rm{r}}}^{(m)}\ge {t}_{(l)}^{(n)}\\ 0 & {\rm{o}}{\rm{t}}{\rm{h}}{\rm{e}}{\rm{r}}{\rm{w}}{\rm{i}}{\rm{s}}{\rm{e}}\end{array}$$

14 $${P}_{rad}^{(m,n)}=[\begin{array}{cc}max({p}_{(l)}^{(m,n)}) & {\rm{i}}{\rm{f}}\,{d}_{min}^{(m,n)} < {d}_{th}\\ 0 & {\rm{o}}{\rm{t}}{\rm{h}}{\rm{e}}{\rm{r}}{\rm{w}}{\rm{i}}{\rm{s}}{\rm{e}}\end{array}$$

15 $${d}_{min}^{(m,n)}=min({d}_{(k,l)}^{(m,n)})$$

### Embers

One of the most prominent modes of propagation are those generated by embers during a wildfire. The embers provide significant amount of complexity to understanding wildfires, as they tend to travel farther downstream than the actual fire front, thereby resulting in multiple fire fronts. The unpredictability and capacity of embers for destruction is a major concern. Ember-driven fire ignitions are heavily influenced by a number of factors such as wind direction (*θ*), wind speed (*v*_*w*_), ember size and shape, among others, which are difficult to account for deterministically. In this study, the effect of embers in regards to possible ignition is modeled using Eq. 16, where ${P}_{acc}^{(i,j)}$ is the relative probability of access for embers and ${g}^{(i,j)}(.):{\mathbb{R}}\mapsto [0,\,1]$ is the probability distribution function between nodes *i* and *j*, given by a distribution function *S* (Eq. 17). The distribution is uniquely defined for each (*i*,*j*) node pair interaction as a function of volume of source node *i* (${V}_{n}^{(i)}$), the distance between nodes *i* and *j* (*d*^(*i*,*j*)^), and wind speed (*v*_*w*_). Details on the ember distribution function are discussed in Section 4 of the SI text.

16 $${P}_{ember}^{(i,j)}={P}_{acc}^{(i,j)}.{F}_{cc}^{(i,j)}.{g}^{(i,j)}$$

17 $${g}^{(i,j)}=S({V}_{n}^{(i)},{d}^{(i,j)},{v}_{w});$$

The probability of access (${P}_{acc}^{(i,j)}$) is an indication of the ease with which an ember can ignite a way based on its design, which may include material properties of way^52^, layout design^54^ and other factors. Relative probabilities are assigned to ways based on their functional classification (Table S5).

## Electronic supplementary material

Supplementary Material

## References

[1] Whitlock C (2004). Land management: Forests, fires and climate. Nature.

[2] Editorial. Spreading like wildfire. *Nature Climate Change* **7**, 755 (2017).

[3] Editorial. Natural events and unnatural disasters. *Nature Plants* **3** (2017). 10.1038/nplants.2017.124 28770822

[4] Westerling AL, Hidalgo HG, Cayan R, Swetnam TW (2006). Warming and earlier spring increase western u.s. forest wildfire activity. Science.

[5] Schoennagel T (2017). Adapt to more wildfire in western north american forests as climate changes. Proceedings of National Academy of Science.

[6] Jolly WM (2015). Climate-induced variations in global wildfire danger from 1979 to 2013. Nature Communications.

[7] Dennison PE, Brewer SC, Arnold JD, Moritz MA (2014). Large wildfire trends in the western united states. Geophysical Research Letters.

[8] Canada, N. R. The state of canada’s forests. Tech. Rep., Canadian Forest Service, Ottawa, Canada (2016).

[9] Stephens SL (2013). Land use. managing forests and fire in changing climates. Science.

[10] Calkin, D. E., Cohen, J. D., Finney, M. A. & Thomson, M. P. How risk management can prevent future wildfire disasters in the wildland-urban interface. *Proceedings of National Academy of Science* **111** (2014). 10.1073/pnas.1315088111 PMC3896199 24344292

[11] Moritz MA (2014). Learning to coexist with wildfire. Nature.

[12] North MP (2015). Environmental science. reform forest fire management. Nature.

[13] Arno, S. F. & Brown, J. K. Overcoming the paradox in managing wildland fire. *Western Wildlands (Montana Forest and Conservation Experiment Station*, *Missoula*, *MT)* 40–46 (1991).

[14] Cohen, J. & Stratton, R. D. Home destruction examination: Grass valley fire, lake arrowhead, ca. Tech. Rep. R5-TP-026b, US Forest Service, Portland, OR, USA (2008).

[15] Rothermel, R. C. A mathematical model for predicting fire spread in wildland fuels. Tech. Rep. Tech. Rep. INT-1 15, U.S. Department of Agriculture, Intermountain Forest and Range Experiment StationResearch (1972).

[16] Knight I, Coleman J (1993). A fire perimeter expansion algorithm based on huygens’ wavelet propagation. International Journal of Wildland Fire.

[17] Andrews, P. L. Behaveplus fire modeling system: past, present, and future. BehavePlus fire modeling system: past, present, and future (Bar Harbor, Maine, USA, 2007).

[18] Finney, M. A. An overview of flammap fire modeling capabilities. Proceedings of the Fuels Management-How to Measure Success, 213–220 (Portland, Ore, USA, 2006).

[19] Stratton, R. D. Guidance on spatial wildland fire analysis: models, tools, and techniques. Tech. Rep. General Technical Report RMRS-GTR-183, U.S. Department of Agriculture, Forest Service, Rocky Mountain Research Station, Ft. Collins, CO, USA (2006).

[20] Finney, M. A. Farsite: fire area simulator–model development and evaluation. Tech. Rep. Tech. Rep. RMRS-RP-4, U.S. Department of Agriculture, Forest Service, Rocky Mountain Forest and Range Experiment Station, Ogden, Utah, USA (1998).

[21] Finney MA, Grenfell IC, McHugh CW (2010). A method for ensemble wildland fire simulation. Environmental Modeling and Assessment.

[22] Altintas, I. *et al*. Wifire: A real- time cyberinfrastructure for wildfire sensing and prediction. Large Wildland Fires Conference, 19–22 (Missoula, MT, USA, 2014).

[23] Albini, F. A. Spot fire distance from burning trees-a predictive model. Tech. Rep. Gen. Tech. Rep. INT-56, U.S. Department of Agriculture, Forest Service, Intermountain Forest and Range Experiment Station, Ogden, UT, USA (1979).

[24] Rochoux MC, Ricci S, Lucor D, Cuenot B, Trouvé A (2014). Towards predictive data-driven simulations of wildfire spread. part i: Reduced-cost ensemble kalman filter based on a polynomial chaos surrogate model for parameter estimation. Natural Hazards and Earth System Sciences.

[25] McGrattan, K. *et al*. Fire dynamics simulator, user’s guide. Tech. Rep., National Institute of Standards and Technology, Gaithersburg, MD (2013).

[26] Finney MA (2002). Fire growth using minimum travel time methods. Canadian Journal of Forest Research.

[27] Stepanov A, Smith JM (2012). Modeling wildfire propagation with delaunay triangulation and shortest path algorithms. European Journal of Operational Research.

[28] Hajian M, Melachrinoudis E, Kubat P (2016). Modeling wildfire propagation with the stochastic shortest path: A fast simulation approach. Environmental Modeling and Software.

[29] Sekizawa A (2000). Information system for supporting fire-fighting activities based on real time fire spread simulation. Proceedings of Institute of Social Safety Science.

[30] Tsujihara O, Terada K, Sawada T (2005). Development of simulation system of spreading fire occurring simultaneously in many places in an earthquake using petri-net. Journal of Applied Computing in Civil Engineering.

[31] USDA & USDI. Protecting people and natural resources: a cohesive fuels treatment strategy. us healthy forests and rangelands plan. Tech. Rep., USDA and USDI (2006).

[32] Mollison D (1977). Spatial contact models for ecological and epidemic spread. Journal of the Royal Statistical Society.

[33] Grassberger P (1983). On the critical behaviour of the general epidemic process and dynamical percolation. Mathematical Biosciences.

[34] Newman, M. E. J. Spread of epidemic disease on networks. *Physical Review E* **66** (2002). 10.1103/PhysRevE.66.016128 12241447

[35] Kretzschmar M, van Duynhoven YTHP, Severijnen AJ (1996). Modeling prevention strategies for gonorrhea and chlamydia using stochastic network simulations. American Journal of Epidemiology.

[36] Haydon DT (2003). The construction and analysis of epidemic trees with reference to the 2001 uk foot-and-mouth outbreak. Proceedings of the Royal Society B.

[37] Riley S (2003). Transmission dynamics of the etiological agent of sars in hong kong: impact of public health interventions. Science.

[38] Klovdahl AS (1985). Social networks and the spread of infectious diseases: the aids example. Social Science and Medicine.

[39] Cohen JD (2004). Relating flame radiation to home ignition using modeling and experimental crown fires. Canadian Journal of Forest Research.

[40] Beverly JL, Bothwell P, Conner JCR, Herd EPK (2010). Assessing the exposure of the built environment to potential ignition sources generated from vegetative fuel. International Journal of Wildfire.

[41] Cohen, J. D. & Stratton, R. Home destruction within the hayman fire perimeter. Tech. Rep. Gen. Tech. Rep. RMRS-GTR-114, U.S. Department of Agriculture, Forest Service, Rocky Mountain Research Station, CO (2003).

[42] Maranghides, A. & Mell, W. A case study of a community affected by the witch and guejito fires. Tech. Rep. Technical Note-1635, National Institute of Standards and Technology, Gaithersburg, MD (2009).

[43] Westhaver, A. Why some homes survived: Learning from the fort mcmurray wildfire disaster. Tech. Rep. ICLR research paper series -no. 56, Institute for Catastrophic Loss Reduction, Toronto, Ontario, Canada (2017).

[44] Team, W. I. W. *Assessing wildfire hazards in the home ignition zone* (Natl. WUI Fire Program. Firewise Communities; NFPA, 2006).

[45] Bredeson, C. *Fire in oakland, california: Billion-dollar blaze*. (Enslow Publishers, Springfield, NJ, USA, 1999).

[46] Pagni PJ (1993). Causes of the 20 october 1991 oakland-hills conflagration. Fire Safety Journal.

[47] California Department of Forestry, C. D. & Protection, F. Oakland/ berkeley hills fire of october 20, 1991, damage assessment survey. Tech. Rep., California Department of Forestry and Fire Protection, Berkeley, CA, USA (1991).

[48] Alexandre PM (2016). Factors related to building loss due to wild res in the conterminous united states. Ecological Applications.

[49] Bonacich P (1987). Power and centrality: A family of measures. American Journal of Sociology.

[50] Freeman LC (1979). Centrality in social networks: Conceptual clarification. Social Networks.

[51] Koo E, Pagni PJ, Weise DR, Woycheese JP (2010). Firebrands and spotting ignition in large-scale fires. International Journal of Wildfire.

[52] Cohen J (2010). The wildland-urban interface fire problem. Fremontia.

[53] Parteners In Protection, P. *FireSmart: protecting your community from wildfire (Second Edition)* (Capital Color Press Ltd. Edmonton, Alberta, 2003).

[54] Cohen JD (2000). Preventing disaster, home ignitability in the wildland-urban interface. Journal of Forestry.

[55] OpenStreetMap contributors. Planet dump retrieved from https://planet.osm.org. https://www.openstreetmap.org (2017).

