# Supplementary material for: Unraveling the Complexity of Wildland Urban Interface Fires
Source: Sci Rep. 2018 Jun 18;8:9315. doi: 10.1038/s41598-018-27215-5 (PMC6006360; doi:10.1038/s41598-018-27215-5)
Supplement: Supplementary file 1 — Supplementary Material [file 41598_2018_27215_MOESM1_ESM.pdf]

SUPPLEMENTARY INFORMATION
Unraveling the Complexity of Wildland Urban Interface Fires

Hussam Mahmoud and Akshat Chulahwat

June 5, 2018

1 Graph Formulation

For all tests in this study, the graphs are developed using GIS data from http://openstreetmap.org. Each way is comprised of multiple nodes, which forms the boundary of its respective way. The edge weights assigned between the nodes define the wildfire propagation probabilities from one node to another. The ways in each community are classified into different categories to recognize ignitable ways. The classification is made into categories shown in table 1 and each of which are further classified into sub-categories to improve the accuracy of the data. Each way classification can have minor categories. For instance, type 'Building' could be further classified into - residential, religious, commercial and others. Similarly, type 'Amenity' could encompass - entertainment, financial, transportation, education and others. An important thing to note is that while all 'Building' types can be considered ignitable, not all sub-classifications of type 'Amenity' would be ignitable. Therefore, classification of ways is an important step as it would govern the input parameters for each way. A detailed list of sub-categories is listed and defined in http://wiki.openstreetmap.org/wiki/Map_Features.

The community layouts derived from openstreetmap.org do not represent the location of discrete vegetation near the houses. To observe the effect of stray vegetation in the neighboring regions of all houses, a sensitivity analysis is conducted using Monte-Carlo simulation for N = 100 iterations. It is observed that for > 95 percentile cases, the mean vulnerability of the community converges to a single value. This suggests that for these layouts even if the location of vegetation around a house is unknown, the total risk of the community does not change drastically when effect of fire intervention is not considered. This can be attributed to the over-redundancy in the fire network. On the other hand, when fire intervention is applied, the effect of presence/absence of vegetation is inherently considered within the framework. The effect of vegetation would affect the fire graph network in general, and for best practice, the stray vegetation would have to be considered as separate nodes in the graph. To circumvent this problem in the future, satellite images can be used in conjunction with a custom image pattern recognition algorithm to identify stray vegetation, which can be overlaid on the Openstreet maps to get an accurate representation of the community layout.

Table 1: Classification of ways based on their ignition capacity

Type	Landuse	Building	Amenity	Natural area	Leisure	Shop	Office	Route	Others
Ignition	Some	All	Some	All	All	All	All	None	None

Three layouts are tested in this study - (a) Hacienda Heights (b) Oakland (O_I) and (c) Oakland (O_{II}). The details of the formulated graph for each layout are shown in table 2. The fuel content percentage I of each community is measured as the ratio of the total ignitable area to the total analysis area (Eq. 1). $N_{\mathcal{W}}$ is the total number of ways, $A_w^{(m)}$ is the area of way m and A^* is the total area of the region used for analysis.

$$I = \frac{\sum_{m=1}^{N_{\mathcal{W}}} A_w^{(m)}}{A^*} \tag{1}$$

Table 2: Details for each community layout

Location	Total Nodes	Total Ways	Fuel Density
Hacienda heights	1304	156	16.37%
Oakland (O_I)	7445	636	12.82%
Oakland (O_{II})	9326	845	18.03%

2 Convection Model

A simplified convection model is used in this study, which utilizes the concept of flame height (Eq. 2) and flame angle (Eq. 3) to determine maximum convection distance (Eq. 4). The flame angle is mapped linearly to wind speed such that $v_w^{min} \rightarrow \theta_f = 90^o$ and $v_w^{max} \rightarrow \theta_f = 30^o$. The parameters for convection model are described in table 3. The current convection model does not account for all mechanisms involved in convective heat transfer. Currently, there exists several other empirical models on convection, which have been calibrated from previous wildfire case studies. These models could provide a suitable alternative.

$$h_f^{(i)} = \alpha.h^{(i)} \tag{2}$$

$$\theta_f = m.v_w + b \tag{3}$$

$$d_{conv}^{max} = h_f^{(i)}/tan(\theta_f) \tag{4}$$

Table 3: Convection model parameter values

Parameter	Definition	Value	Reference
α	Flame height constant	1.3	-
m	slope	-2.065	-
b	constant	90°	-
v_w^{min}	Minimum wind velocity	0 mph	-
v_w^{max}	Maximum wind velocity	65 mph	-

3 Radiation Model

3.1 View factors calculation

Sparrow [1] reduced the double area integral of view factor formula to the contour double integral formula (CDIF), using the Stokes' theorem, such that when the contours of the source P and target surface Q are divided into segment vectors $g^{(k)\in[1,2,...,n_k]}$ and $g^{(l)\in[1,2,...,n_l]}$, the view factor can be approximated by the discretization expression [2] given in Eq. 5.

$$F_{P-Q} = \frac{1}{2\pi A_P} \oint_{\Gamma_Q} \oint_{\Gamma_P} ln(S) ds_P.ds_Q \tag{5}$$

A_P is the area of surface P, Γ_P and Γ_Q are the contours bounding surfaces P and Q, ds_P and ds_Q are differential length vectors and S is the distance between differential elements of surface P and Q. The discretization equation is further simplified as shown in Eq. 6, where the discretization of surface Q is replaced by the mid-point of surface Q such that $S_m^{(k)}$ is given by the distance between k^{th} differential element of surface P and mid-point of surface Q. For the purpose of identifying pilot ignition, only a point on the surface needs to be ignited; therefore, discretization of the target surface is not required. The formulation is solved using a 10-point Gauss quadrature scheme for all calculations, as it provides sufficient accuracy [3].

$$F_{P-Q} = \frac{1}{2\pi A_P} \sum_{k=1}^{n_k} ln(S_m^{(k)}).g^{(k)} \tag{6}$$

3.2 Radiation matrix formulation

To develop the local radiation matrix between way m and n, such that m is the source and n is the destination way, the following steps are involved:

- Check numbering of ways: $A_{(p)} = \left\{ \sum_{v=1}^{N_{\mathcal{W}(k)}-1} (x_{(p)}^{(v+1)} - x_{(p)}^{(v)}).(y_{(p)}^{(v+1)} + y_{(p)}^{(v)}) | p \in [1, N_{\mathcal{W}}] \right\}$. If $A_{(p)} > 0$ anti-clockwise numbering and $A_{(p)} < 0$ clockwise numbering for way p.
- Find midpoint of all surfaces of a way: $xm_{(p)}^{(v)} = 0.5(x_{(p)}^{(v+1)} + x_{(p)}^{(v)})$ and $ym_{(p)}^{(v)} = 0.5(y_{(p)}^{(v+1)} + y_{(p)}^{(v)})$.
- Find normal for all surfaces of a way: $\hat{n}_{(p)}^{(v)} = [\hat{n}x_{(p)}^{(v)} \hat{n}y_{(p)}^{(v)}] = A.[-dy_{(p)}^{(v)} \quad dx_{(p)}^{(v)}]$, where $dx_{(p)}^{(v)} = x_{(p)}^{(v+1)} - x_{(p)}^{(v)}$ and $dy_{(p)}^{(v)} = y_{(p)}^{(v+1)} - y_{(p)}^{(v)}$.
- Draw line segments joining the midpoints of all k surfaces of way m with l surfaces of way n to obtain their respective vector equation: $xr_{(k,l)}^{(m,n)} = xm_{(k)}^{(m)} - xm_{(l)}^{(n)}$ and $yr_{(k,l)}^{(m,n)} = ym_{(k)}^{(m)} - ym_{(l)}^{(n)}$.
- Check all line segments generated from the source way for intersection with self for all values of p: $ts_{(k,l)}^{(m,n)} = \frac{[xr_{(k,l)}^{(m,n)} \quad yr_{(k,l)}^{(m,n)}].[\hat{n}x_{(k)}^{(m)} \quad \hat{n}y_{(k)}^{(m)}]}{-[xr_{(p,l)}^{(m,n)} \quad yr_{(p,l)}^{(m,n)}].[\hat{n}x_{(p)}^{(m)} \quad \hat{n}y_{(p)}^{(m)}]} \quad \forall \quad p \in \mathcal{F}_{(m)}$. If $ts_{(p,l)}^{(m,n)}$ lies on the source polygon $Ms_{(k,l)}^{(m,n)} = 1$, else 0.
- Find the intersection point(s) for all line segments with each face of the destination way for all values of q: $td_{(k,q)}^{(n,m)} = \frac{[xr_{(k,l)}^{(n,m)} \quad yr_{(k,l)}^{(n,m)}].[\hat{n}x_{(l)}^{(n)} \quad \hat{n}y_{(l)}^{(n)}]}{-[xr_{(k,q)}^{(n,m)} \quad yr_{(k,q)}^{(n,m)}].[\hat{n}x_{(q)}^{(n)} \quad \hat{n}y_{(q)}^{(n)}]} \quad \forall \quad q \in \mathcal{F}_{(n)}$. If $ts_{(k,q)}^{(n,m)}$ lies on the destination polygon $Md_{(k,l)}^{(m,n)} = 1$, else 0.
- Accessibility matrix, which represents the surfaces of destination way (n) accessible by each surface of source way (m), is defined as - $Ac^{(m,n)} = Ms_{(k,l)}^{(m,n)} \wedge Md_{(k,l)}^{(m,n)}$.
- The individual heat flux of each surface is updated using the accessibility matrix as $q_{(k,l)}^{(i,j)} = Ac^{(i,j)}.q_{(k,l)}^{(i,j)}$

3.3 Radiation Ignition Parameters

The parameter values used in this study are shown in table 4.

Table 4: Radiation model parameter values

Parameter	Definition	Value	Reference
T_a	Ambient temperature of surroundings	300 K	[4]
T_f	Flame temperature	1000 K	[4]
$Q_{cr}^{(n)}$	Critical flux required for ignition (for wood)	13.1 kW/m^2	[4]
$FTP^{(n)}$	Flux time product (for wood)	13500 $kW/m^2.s$	[4]
c	Constant (obtained from experimental data)	-1.828	[4]
d_{th}	Radiation threshold distance	30 m	[4]
$t_{r,min}$	Lower bound on residence time	45 s	-
$t_{r,max}$	Upper bound on residence time	240 s	-
λ	Standard deviation	65 s	-
$\epsilon_{(k)}^{(m)}$	Emissivity (for wood)	0.84	-
σ	Stefan-Boltzmann constant	5.67x10$^{-8}Wm^{-2}K^{-4}$	-

4 Ember Model

The ember model developed by Martin and Hillen [5] is utilized to obtain the nodal ember probability distribution (S). The model is based on the concept of birth-jump processes, which has been studied in context of wildfire spotting [6]. The ignition probability of an ember can be attributed to the amount of mass present in an ember upon landing [5]. Eq. 7 gives the landing mass of an ember (M), which is incorporated in a piecewise linear ignition function (Eq. 8) to obtain the ignition probability S.

$$M(i, x, v_w) = N_e^{(i)} \lambda a \left(\exp^{-\lambda |v_t| \frac{x}{w}} \right) \left(\frac{2}{3} m_{max}^{\frac{3}{2}} - \frac{2\kappa x m_{max}^{0.5}}{v_w} + \left(\frac{4}{3} \frac{\kappa x}{v_w} \right)^{1.5} \right) \tag{7}$$

$$S(i, x, v_w) = E(M(i, x, v_w)) = \begin{cases} \frac{M(i,x)}{m_{min}} & \text{if } M(i, x) \leq m_{min} \\ 1 & \text{if } M(i, x) > m_{min} \end{cases} \tag{8}$$

$N_e^{(i)}$ is the number of embers generated by node i and given by Eq 9 as a function of its fuel volume ($V^{(i)}$). N_e^* and V^* are scaling parameters. The fuel volume of a node ($V_n^{(i)}$) is the amount of ignitable fuel present at a node and it is calculated assuming equal distribution between all nodes of a way. It is given by Eq. 10, where $V_w^{(m)}$ is the total fuel volume of way m (ignited or source way), $W_{(m)}$ is the node set of way m and $N_{(m)}$ is the total number of nodes. In this study, stray vegetation around the ways is not marked explicitly in the community layout; therefore, the entire volume of ways is considered as fuel volume to offset the absence of vegetation and to keep the analysis on the conservative side. For best practice, the fuel volume at each node would have to be considered explicitly based on nature of the way.

$$N_e^{(i)} = V_n^{(i)} \left(\frac{N_e^*}{V^*} \right) \tag{9}$$

$$V_n^{(i)} = \left(\frac{V_w^{(m)}}{N_{(m)}} \right)_{i \in \mathcal{W}_{(m)}} \tag{10}$$

To calibrate the ember distribution model, previous experimental investigations on ember spotting are used [7, 8, 9]. The experiments comprised of burning individual tree samples under constant wind and collecting generated embers to determine their respective mass distribution. N^* is the number of embers collected and V^* is the volume of tree used in the experiment (Eq. 11), which is calculated assuming a cylindrical configuration. r^* and h^* are the radius and height of tree used. The values of all parameters are shown in table 6.

$$V^* = \pi (r^*)^2 h^* \tag{11}$$

Figure 1 shows the effect of 3 key parameters - Fuel volume of node, distance between nodes and wind speed, on the ember distribution obtained from the discussed formulation. The ember distribution model presented above is only one such model. There exists several other possibilities based on the underlying assumptions considered in formulating the model. Some of the possibilities are presented in [6]. The scope of this paper is limited to testing the efficacy of the graph model only. Hence, only one configuration of ember model is considered. For future work, other possibilities of ember models can be tested to inquire the sensitivity of the graph model to micro factors.

One of the biggest limitations of the discussed ember model is that it is highly sensitive to changes in wind speed. In actual wildfires, severe fluctuations in the local wind field occurs due to change in temperature gradient with time. As a result, embers in most cases are observed to fly in different directions than the dominant wind direction. A prominent example of this phenomena is the generation of fire vortices, which are observed in high intensity wildfires. These vortices tend to distribute embers in all directions irrespective of the dominant wind direction over the area.

Table 5: Relative access probabilities for different type of ways

Landuse	Building	Natural area	Leisure	Shop	Office
1.0	0.90	1.0	0.80	0.85	0.70

Table 6: Ember model parameter values

Parameter	Definition	Value	Reference
m_{min}	Minimum ember mass observed	0.001 kg	[7]
m_{max}	Maximum ember mass observed	0.004 kg	[7]
a	Mass distribution parameter	7.91	[7]
v_t	Ember terminal velocity	-4.3 m/s	[10]
κ	Ember combustion rate	0.00005 m/s^2	[5]
λ	Ember decay rate	0.01 m/s^2	[5]
N^*	Reference number of embers	100	[7]
r^*	Reference radius	2 m	[7]
h^*	Reference height	5.2 m	[7]

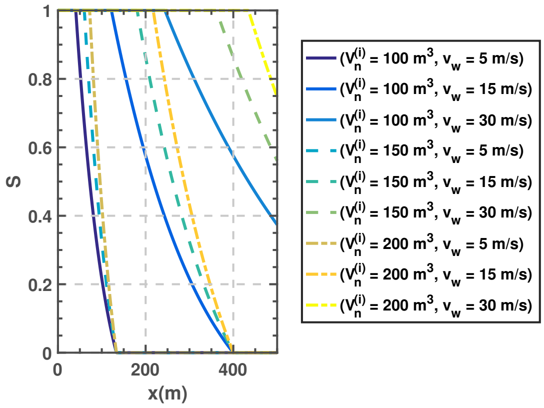

Figure 1: Ember ignition probabilities as a function of distance x for different values of volume $V_n^{(i)}$ and wind speed v_w

In the current model, these changes to the wind field have not been accounted for. However, the graph model has been formulated in a way such that these changes can be effectively included in future studies. By coupling the ember model with a computational fluid dynamics model the local changes in wind can be tracked and used to update the ember model at each time step.

5 Most probable paths (MPPs)

The most probable paths are defined as the product of edges that give the maximum probability of fire reaching a certain point. To identify MPPs, a combination of two algorithms are used (1) Dijkstra's algorithm [11] and (2) Yen's algorithm [12]. The former is used to identify the geodesic path and the latter is used to identify K geodesic paths in the graph. To utilize these algorithms the weight of edges are modified as $W = log(P_{tr})$, where P_{tr} is the edge weight of original graph $\mathcal{G}$. The maximum product problem is converted into a minimum sum problem. Once K shortest paths are calculated, the total weight of each path is reverted to obtain the total probability of each MPP. Each way in the community is a collection of nodes, and can be essentially represented by a complete graph. Since internal edge weights are always unity (conduction dominance inside ways), Yen's algorithm could provide superfluous paths. The algorithm is modified to account for this anomaly.

6 Computational resources and observations

All analysis in this study are performed on an Intel Xeon E5-2620 v3 12-core processor. The computation is divided into 2 phases - (a) Graph formulation of community (phase-I) (2) Calculation of vulnerability for each way (phase-II). The first phase is conducted by CPU parallel computing, while the latter without. The run times for the different community layouts are shown below in Table 7. An important point to note is that the run times shown below are only for one iteration. For certain steps, such as evaluation of fire intervention efficiency, multiple iterations have to be performed, which further increases the computation time. For phase II different iterations are required, but the computation time was kept manageable for all analysis ($<$ 2hrs).

Table 7: Computational details for the test cases

Location	Total Nodes	Total Ways	Phase-I	Phase-II
Hacienda heights	1304	156	300 ± 20 secs	15 ± 5 secs
Oakland (O_I)	7445	636	720 ± 20 secs	65 ± 5 secs
Oakland (O_{II})	9326	845	660 ± 20 secs	95 ± 5 secs

Phase-I includes formulation of the graph by calculating the weights of each edge. The weights are calculated by combining the results from the heat transfer models of Conduction, Convection, Radiation and Embers. About 95% of the computation time for phase-I is related to the radiation model. Replacing the model by a suitable approximation model can result in significant reduction in computation. Parallel computing using a GPU processor was also tested, however, it was found to be impractical due to large data overhead.

7 Eigenvector and Bonacich Centrality

The eigenvector centrality ($C_e^w(m)$) is calculated as the mean of nodal eigenvector centralities ($C_e^n(v)$) of all nodes z that belong to nodal set $\mathcal{W}_{(m)}$ (Eq. 12). The Bonacich centrality is calculated in the same way as well.

$$C_e^w(m) = \frac{\sum^{z \in \mathcal{W}_{(m)}} C_e^n(z)}{N_{\mathcal{W}_{(m)}}} \tag{12}$$

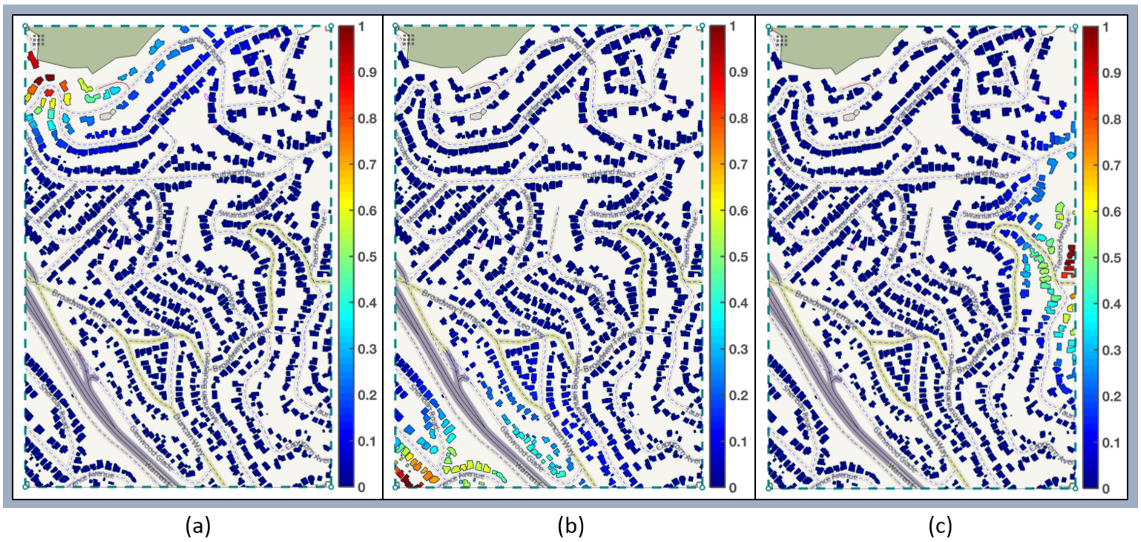

Figure 2: Eigenvector centrality of region O_{II} for wind directions (a)$\theta = 300^o$ (b)$v_w = 60^o$ (c)$v_w = 180^o$ (©OpenStreetMap contributors [13])

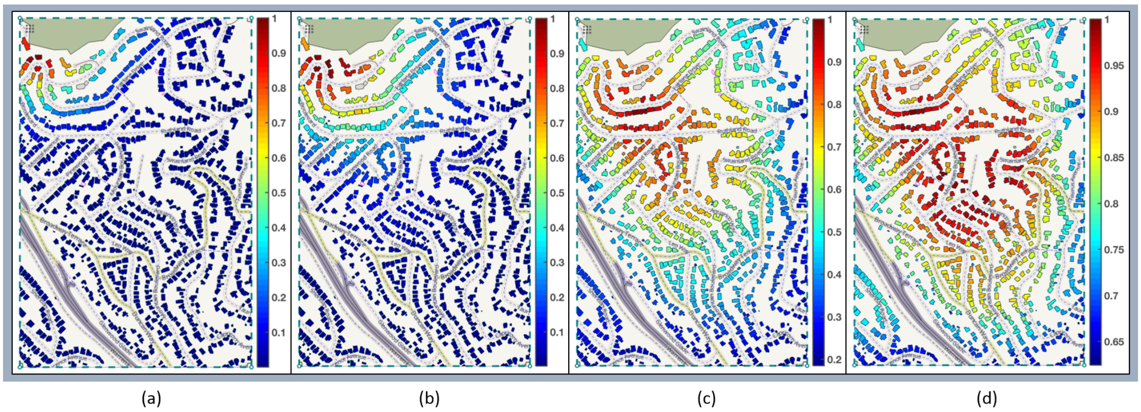

Figure 3: Bonacich centrality of region O_{II} for (a)$\beta = 0.75$ (b)$\beta = 0.50$ (c)$\beta = 0.25$ (d)$\beta = 0.10$ (©OpenStreetMap contributors [13])

8 Degree Centrality

For a directed graph, the indegree ($C^n_{d,i}(v)$) of each node v is calculated as the sum of all edge weights incident on the node, and outdegree ($C^n_{d,o}(v)$) as the sum of all edge weights originating from node v. The total degree ($C^{n*}_d(v)$) is calculated as the sum of indegree and outdegree. To calculate degree centralities for way m the nodal degree centralities is readjusted first, as shown in Eqs. 13 and 14. When calculating way degree centralities, the edges corresponding to internal propagation are removed as they provide no relevant information. The way centralities are then calculated as the sum of adjusted nodal degree centralities (Eq. 15) for all z nodes that belong to the nodal set of way m, as given by Eq. 16.

$$C^{n*}_{d,i}(v) = (C^n_{d,i}(v) - N_{\mathcal{W}_{(m)}})_{\{v \in \mathcal{W}_{(m)}\}} \tag{13}$$

$$C^{n*}_{d,o}(v) = (C^n_{d,o}(v) - N_{\mathcal{W}_{(m)}})_{\{v \in \mathcal{W}_{(m)}\}} \tag{14}$$

$$C^{n*}_d(v) = C^{n*}_{d,i}(v) + C^{n*}_{d,o}(v) \tag{15}$$

$$C^{w*}_d(m) = \sum^{z \in \mathcal{W}_{(m)}} C^{n*}_d(z) \tag{16}$$

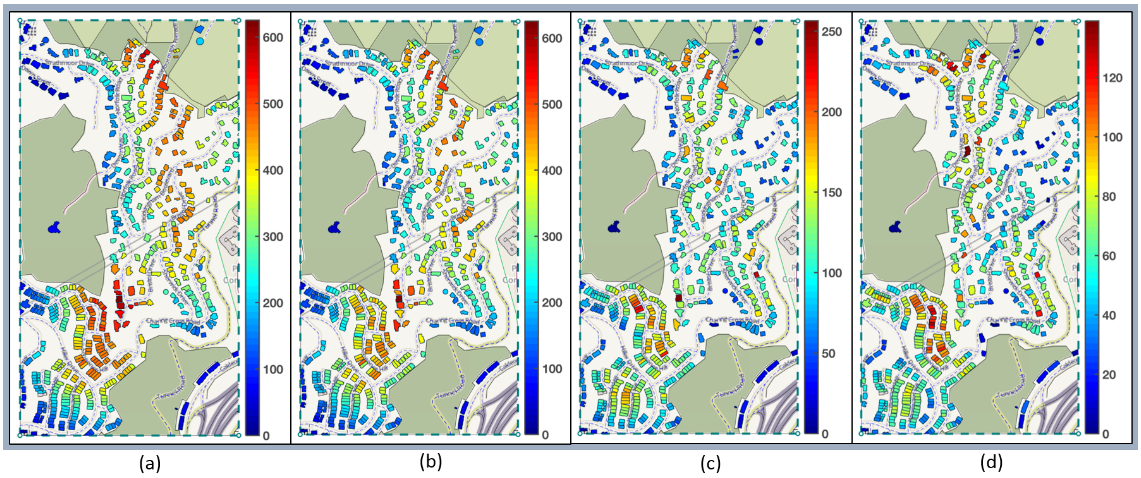

Figure 4: Degree centrality of region O_I for wind speeds (a)$v_w = 29.058 m/s$ (b)$v_w = 10 m/s$ (c)$v_w = 5 m/s$ (d)$v_w = 1 m/s$ (©OpenStreetMap contributors [13])

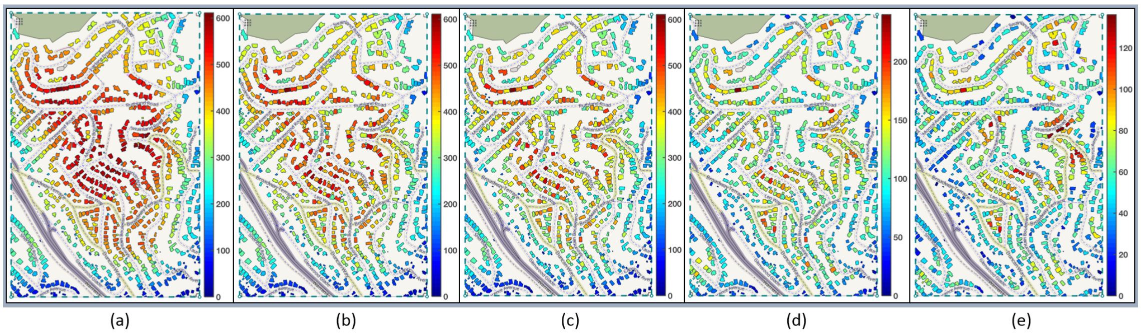

Figure 5: Degree centrality of region O_{II} for wind speeds (a)$v_w = 29.058 m/s$ (b)$v_w = 12.5 m/s$ (c)$v_w = 10 m/s$ (d)$v_w = 5 m/s$ (e)$v_w = 1 m/s$ (©OpenStreetMap contributors [13])

Table 8: Kendall rank correlation (τ) for degree centrality of region O_I at different wind speeds

Wind Speed	29.058 m/s	10 m/s	5 m/s	1 m/s
29.058 m/s	1.0	0.045	0.035	0.035
10 m/s	0.045	1.0	0.04	0.010
5 m/s	0.035	0.04	1.0	0.067
1 m/s	0.035	0.010	0.067	1.0

Table 9: Kendall rank correlation (τ) for degree centrality of region O_{II} at different wind speeds

Wind Speed	29.058 m/s	10 m/s	5 m/s	1 m/s
29.058 m/s	1.0	0.75	0.543	0.381
10 m/s	0.75	1.0	0.697	0.464
5 m/s	0.543	0.697	1.0	0.707
1 m/s	0.381	0.464	0.707	1.0

9 Transitivity

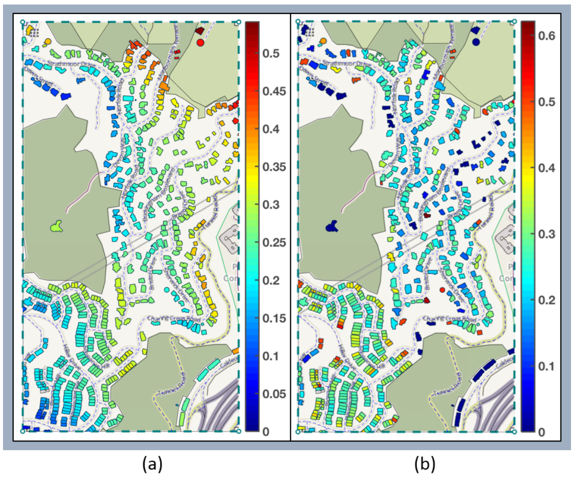

Figure 6: Transitivity of region O_I for (a)$v_w = 29.058m/s$ and (b)$v_w = 0m/s$ (©OpenStreetMap contributors [13])

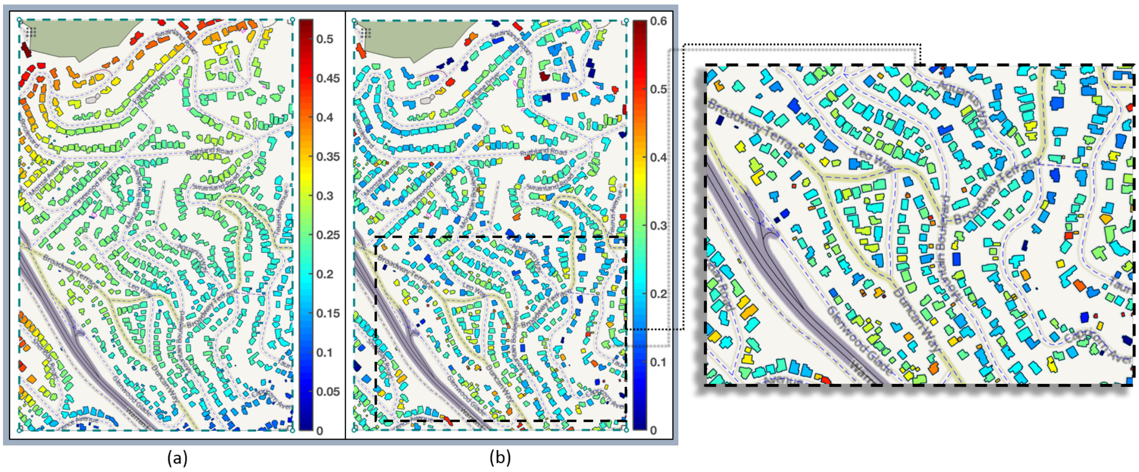

Figure 7: Transitivity of region O_{II} for (a)$v_w = 29.085m/s$ and (b)$v_w = 0m/s$. The magnified snapshot shows certain ways with low area to exhibit high transitivity values (©OpenStreetMap contributors [13])

10 Intervention Framework

- The proposed graph model is implemented to obtain a directed cyclic graph ($\mathcal{G}$) for the community layout.
- An appropriate value for μ is defined for the given community to reflect fire mitigation capacity in terms of percentage of ways.
- Based on the strength of fire mitigation chosen (μ), a percentage of the total ignitable ways present in the community $\mu.N_{\mathcal{W}}$ are chosen at random, assuming a uniform distribution, to create the set $\{\mathcal{W}_M | \mathcal{S} \notin \mathcal{W}_M\}$, where $\mathcal{S}$ is the set of source nodes.
- The inflow and outflow for each node of the ways in the formulated set $\mathcal{W}_M$ are altered to modify the original graph ($\mathcal{G}$). Equation 17 represent the change in indegree and outdegree of each node for the selected ways.

$$a_{(v,j)} = \alpha . a_{(v,j)} \quad a_{(j,v)} = \beta . a_{(j,v)} \quad \forall \quad \{v \in \mathcal{W}_M^{(l)} | l = 1 : N_{\mathcal{W}_M}, j = 1 : n\} \tag{17}$$

- α and β are mitigation scaling factors that are assumed to be 0.10 and 0.75. α represents the scaling factor for outflow from node v, which would be affected by factors such as sprinkler systems and others. β represents the scaling factor for inflow to node v, which would include fire-proof paint and barriers, firefighters and others. The former value is assumed based on the fire mitigation capacity of sprinklers [14].

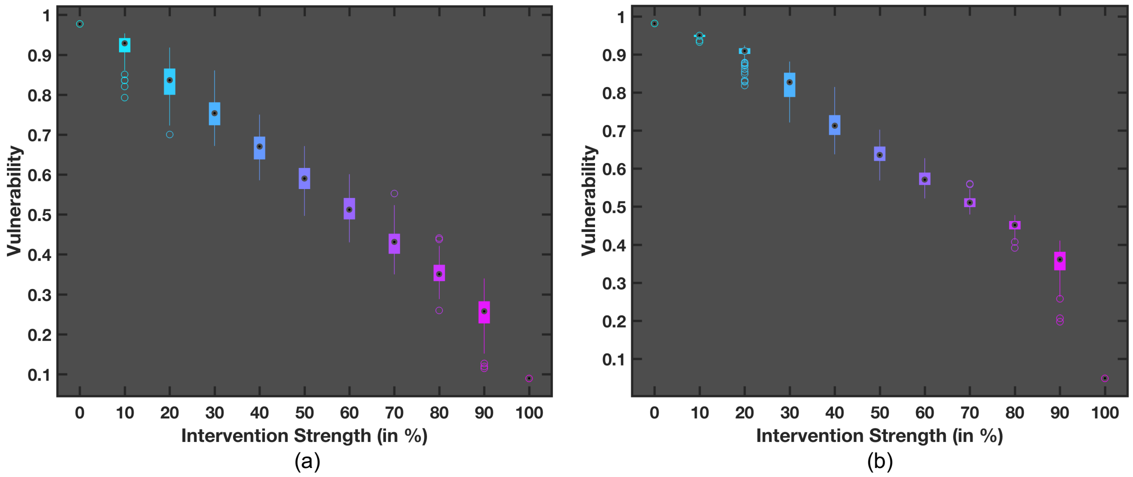

Figure 8: Vulnerability distributions obtained for $N = 100$ iterations at different intervention strengths (μ) for (a) region O_I (b) region O_{II}. The lower and upper edges of the box correspond to the 25th and 75th percentile, the center dot represents the median and circular markers represent the outliers

11 Source Selection Framework

- Initial source location is selected.
- A temporary second source is placed at ignitable way $m \in N_{\mathcal{W}}$.
- Probability of ignition due to wildland nodes for every way (Eq. 18) is calculated, as shown in Fig. 2 in main text. For each node of a way the probability of ignition is calculated as the maximum of ignition probabilities from all boundary nodes of wildland (Ω).

$$P_i^{(s)} = \max\left(g^{(b,s)}\right) = \max\left(S(d^{(b,s)}, \phi^{(b,s)}, \theta)\right)_{\{b \in \Omega, s \in \mathcal{S}\}} \tag{18}$$

- Total vulnerability of community (V) is calculated by evaluating the individual way vulnerabilities and calculating the mean for the community ($V(m)$).
- The way that results in the maximum vulnerability of the community is added as the next source location.
- The above process is repeated for l number of iterations to obtain l source locations.
- The process is stopped when increment in total vulnerability is reduced below a threshold percentage ($< 1\%$).

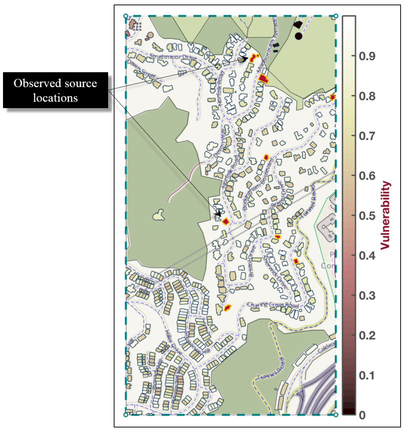

Figure 9: Vulnerability distribution of region O_I for source locations observed during 1991 Oakland fire (Reconstructed from [15]) (©OpenStreetMap contributors [13])

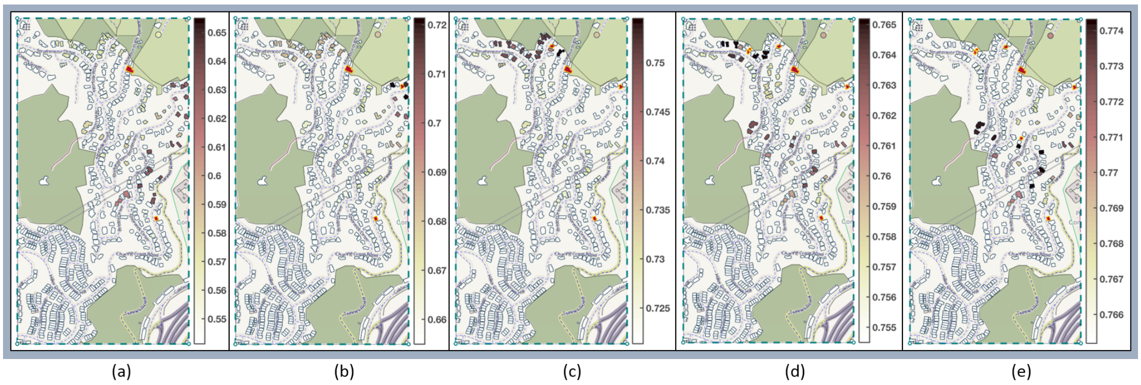

Figure 10: Total community vulnerability shown at each way when that specific way is considered as a source. With each iteration the way which results in maximum community vulnerability is added to the list of sources. The figures show the change in community vulnerability for region O_I as sources are added for iteration (a)$i = 1$ (b)$i = 2$ (c)$i = 3$ (d)$i = 4$ (e)$i = 5$ (©OpenStreetMap contributors [13])

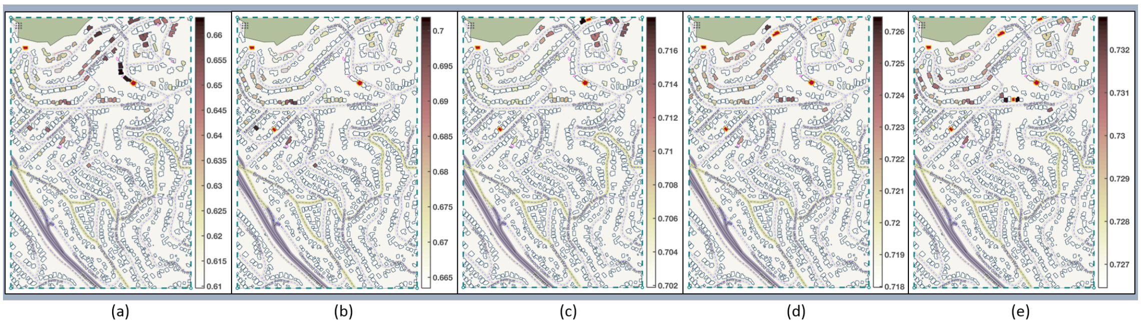

Figure 11: Total community vulnerability shown at each way when that specific way is considered as a source. With each iteration the way which results in maximum community vulnerability is added to the list of sources. The figures show the change in community vulnerability for region $O_I I$ as sources are added for iteration (a)$i = 1$ (b)$i = 2$ (c)$i = 3$ (d)$i = 4$ (e)$i = 5$ (©OpenStreetMap contributors [13])

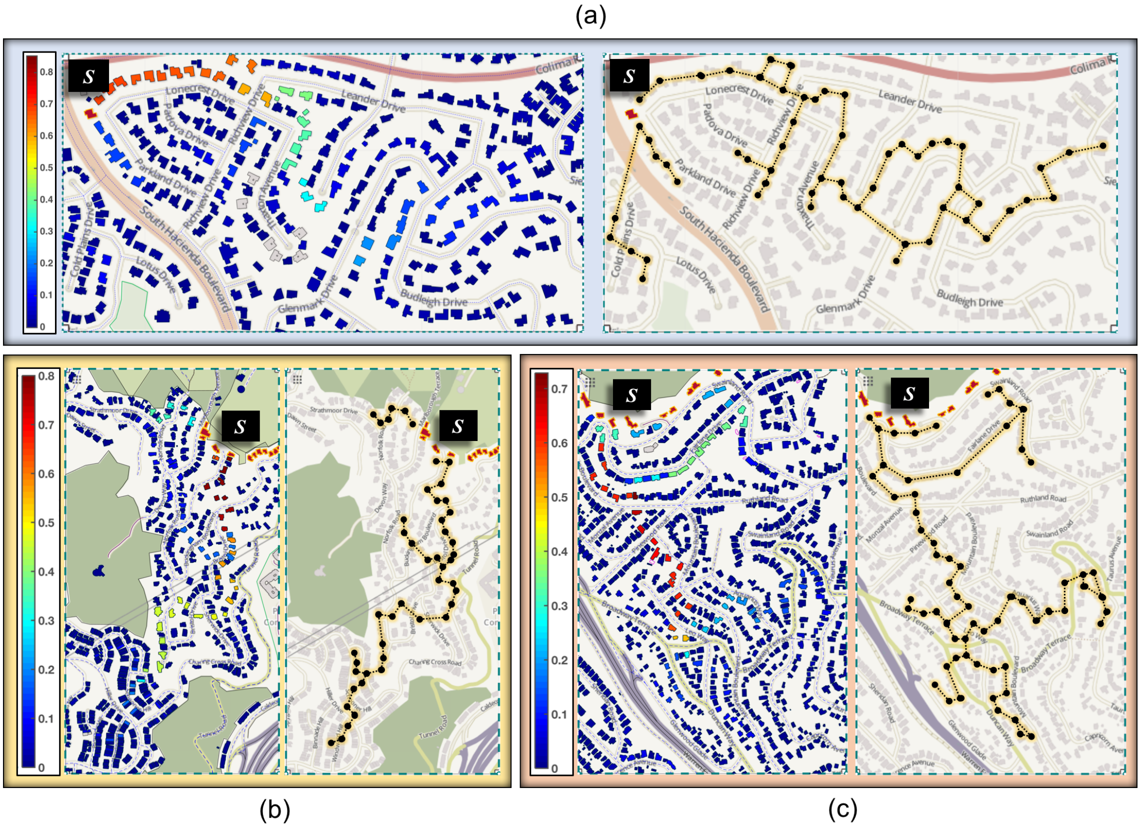

Figure 12: Maximum probability flow paths at wind speed $v_w = 29.058 m/s$, considering only short range propagation modes - radiation and convection, for regions (a) Hacienda Heights ($v_w = 300^o$) (b) Oakland region O_I (c) Oakland region O_{II} (©OpenStreetMap contributors [13])

12 Betweenness Centrality

Betweenness centrality is a measure based on the shortest path between nodes [16]. The application of betweenness to wildfire graphs, can determine the pattern of maximum flow paths within a community. However, the traditional definition of betweenness does not suffice in this case. Betweenness centrality measures the connectivity of a graph assuming an unbiased directionality in flow. There are only specific nodes in wildfire network that generate flow. By including other non-source nodes the centrality measure is diluted. The betweenness centrality measure for wildfire propagation in a community can be considered a sub-case of betweenness in a typical graph. To identify the maximum flow paths the normalized centrality (C_b^{n*}) is defined as shown in Eq. 19, where $\sigma^{(s,j)}$ is the shortest number of paths between nodes s and j, $\sigma^{(s,j)}(z)$ is number of those paths passing through node z, $\mathcal{S}$ is the source node set, $N_\mathcal{S}$ is the total number of sources considered for analysis and N^* is the normalization constant (Eq. 20). Since each way is a complete directed graph, ignition of any node would cause ignition of its respective way, therefore, the betweenness of a way ($C_b^{w*}(m)$) is calculated by Eq. 21.

$$C_b^{n*}(z) = \frac{1}{N^*} \sum_{s=1}^{N_\mathcal{S}} \left(\frac{\sum_{i \neq z \neq j} \sigma^{(s,j)}(z)}{\sigma^{(s,j)}} \right)_{\{s \in \mathcal{S}, (z,j) \in \mathcal{V}\}} \tag{19}$$

$$N^* = \frac{(N_\mathcal{S})(N - N_\mathcal{S} - 1)}{2} \tag{20}$$

$$C_b^{w*}(m) = \sum^{z \in \mathcal{W}_{(m)}} C_b^{n*}(z) \tag{21}$$

Notations

Table 10: Parameter definitions

Parameter	Definition
$\mathcal{G}$	Graph of a community
$\mathcal{V}$	Total node set of graph $\mathcal{G}$
$\mathcal{E}$	Total edge set of graph $\mathcal{G}$
A	Adjacency matrix
n	Total number of nodes in graph $\mathcal{G}$
$a_{(i,j)}$	(i,j) entry of adjacency matrix A
$P_{tr}^{(i,j)}$	Probability of fire transfer from node i to j
I	Fuel Density (in %)
$\mathcal{W}_{(k)}$	Node set of way k
$N_{\mathcal{W}}$	Total number of ways in graph $\mathcal{G}$
i	index for source nodes (ignited node)
j	index for target nodes
m	index for ways
$P_{cond}^{(i,j)}$	Probability of fire transfer by conduction from node i to j
$P_{conv}^{(i,j)}$	Probability of fire transfer by convection from node i to j
$P_{rad}^{(i,j)}$	Probability of fire transfer by radiation from node i to j
$P_{ember}^{(i,j)}$	Probability of fire transfer by ember spotting from node i to j
MPP	Most probable path
$\mathcal{M}_{(x)}$	Adjacency list of x MPP
$N_{\mathcal{M}_{(x)}}$	total members in adjacency list $\mathcal{M}_{(x)}$
$P_{MPP}^{(x)}$	Total probability of MPP x
K	Number of shortest paths
$P_m^{(s)}$	Mean probability of K shortest paths for source node s
$P_i^{(s)}$	Probability of ignition for source node s
$N_{\mathcal{S}}$	Number of nodes in set $\mathcal{S}$
$V^{(z)}$	Total vulnerability of node z
μ	Fire intervention strength
$d^{(i,j)}$	Distance between nodes i and j
$d_{conv}^{(i,j)}$	Convection threshold distance
$h_f^{(i)}$	Flame height
θ_f	Flame angle
θ	Wind direction
$F_{cc}^{(i,j)}$	Wind correlation coefficient
$\phi^{(i,j)}$	Angle of edge joining node i to j w.r.t x-axis
k	index for surface of ignited (burning) way
l	index for surface of target way

Table 11: Parameter definitions (contd.)

Parameter	Definition
$\mathcal{F}_{(.)}$	Set of surfaces for way $(.) = m, n$
$q_{(k,l)}^{(m,n)}$	Heat flux due to radiation from surface k of ignited way m to surface l of way n
$vf_{(k,l)}^{(m,n)}$	View factor between surfaces of different ways
$A_{(k)}^{(m)}$	Area of surface k
σ	Stefan Boltzmann constant
$\epsilon_{(k)}^{(m)}$	Emissivity of surface k
T_f	Flame temperature
T_a	Temperature of surroundings (Ambient temperature)
$\Theta_{(k)}^{(m)}$	Inclination of source surface k
$\Theta_{(l)}^{(n)}$	Inclination of target surface l
$d_{(k,l)}^{(m,n)}$	Distance between surfaces k and l
$Rad[m,n]$	Radiation matrix
$q_{(l)}^{(m,n)}$	Total radiation heat flux on surface l
$t_{(l)}^{(m,n)}$	Minimum residence time for flames required for ignition
$FTP^{(n)}$	Flux time product
$Q_{cr}^{(n)}$	Critical flux for ignition
$c^{(n)}$	Constant
$t_r^{(m)}$	Residence time of each surface of ignited way m
$F(.)$	Normal cumulative distribution function
$d_{min}^{(m,n)}$	Minimum distance between all possible node combinations of ways m and n
$P_{rad}^{(m,n)}$	Probability of fire transfer by radiation from way m to n
v_w	Wind speed
$P_{acc}^{(i,j)}$	Probability of access
$S(i, d^{(i,j)}, v_w)$	Ember spotting probability distribution function
$V_n^{(i)}$	Fuel volume of source node i
$C^d(z)^n$	Total degree centrality of node z
$C_{d,i}^n(z)$	Indegree of node z
$C_{d,o}^n(z)$	Outdegree of node z
$C_{d,i}^w(m)$	Indegree of way m
$C_{d,o}^w(m)$	Outdegree of way m
$C_d(m)^w$	Total degree centrality of way m
$C_e^n(z)$	Eigenvector centrality of node z
$C_e^w(m)$	Eigenvector centrality of way m
$T^n(z)$	Transitivity of node z
$T^w(m)$	Transitivity of way m
$C_b^n(z)$	Betweenness centrality of node z
$C_{b*}^n(z)$	Normalized betweenness centrality of node z
$C_{b*}^w(m)$	Normalized betweenness centrality of way m
N^*	Normalization constant for betweenness centrality
η	Fire intervention (mitigation) strategy efficiency

References

[1] E M Sparrow. A new and simpler formulation for radiative angle factors. *Journal of Heat Transfer*, 81:81–87, 1963.

[2] N Kakuta, S Yokoyama, M Nakamura, and K Mabuchi. Estimation of radiative heat transfer using a geometric human model. *IEEE transactions on bio-medical engineering*, 48(3):324–331, 2012.

[3] S Mazumder and M Ravishankar. General procedure for calculation of diffuse view factors between arbitrary planar polygons. *International Journal of Heat and Mass Transfer*, 55(23-24):7330–7335, 2012.

[4] J D Cohen. Relating flame radiation to home ignition using modeling and experimental crown fires. *Canadian Journal of Forest Research*, 34(8):1616–1626, 2004.

[5] J Martin and T Hillen. The spotting distribution of wildfires. *Applied Sciences*, 6:177, 2016.

[6] T Hillen, B Greese, J Martin, and G de Vries. Birth-jump processes, with applications to wildfire spotting. *Journal of Theoretical Biology*, 9:104–127, 2015.

[7] S L Manzello, T G Cleary, J R Shields, and J C Yang. On the ignition of fuel beds by firebrands. *Fire Materials*, 30:77–87, 2006.

[8] S L Manzello, A Maranghides, and W E Mell. Firebrand generation from burning vegetation. *International Journal of Wildfire*, 16:458–462, 2007.

[9] S L Manzello, A Maranghides, J R Shields, W E Mell, and Y Hayashi. Mass and size distribution of firebrands generated from burning korean pine trees. *Fire Materials*, 33:21–31, 2009.

[10] H B Clements. Lift-off of forest firebrands. Technical Report Research Paper SE-159, USDA Forest Service, Southeastern Forest Experiment Station, Forest Service, USDA, Asheville, North Carolina, USA, 1977.

[11] E W Dijkstra. A note on two problems in connection with graphs. *Numerische Mathematik*, 1:269–271, 1959.

[12] J Y Yen. Finding the k shortest loopless paths in a network. *Management Science*, 17(11):712–716, 1971.

[13] OpenStreetMap contributors. Planet dump retrieved from https://planet.osm.org . https://www.openstreetmap.org, 2017.

[14] J Xin and C Huang. Fire risk analysis of residential buildings based on scenario clusters and its application in fire risk management. *Fire Safety Journal*, 62:72–78, 2013.

[15] J P Woycheese. *Brand lofting and propagation from large-scale fires.* PhD thesis, University of California, Berkeley, 2000.

[16] L C Freeman. A set of measures of centrality based upon betweenness. *Sociometry*, 40:35–41, 1977.
